# Spatial Self-Organization of Vegetation Subject to Climatic Stress—Insights from a System Dynamics—Individual-Based Hybrid Model

**DOI:** 10.3389/fpls.2016.00636

**Published:** 2016-05-24

**Authors:** Christian E. Vincenot, Fabrizio Carteni, Stefano Mazzoleni, Max Rietkerk, Francesco Giannino

**Affiliations:** ^1^Biosphere Informatics Laboratory, Department of Social Informatics, Graduate School of Informatics, Kyoto UniversityKyoto, Japan; ^2^Dipartimento di Agraria, Università degli Studi di Napoli Federico IIPortici, Italy; ^3^Environmental Sciences Group, Copernicus Institute of Sustainable Development, Utrecht UniversityUtrecht, Netherlands

**Keywords:** simulation, mechanistic, shift, climate, self-organization, spatial patterns, agent-based modeling, stock-and-flow

## Abstract

In simulation models of populations or communities, individual plants have often been obfuscated in favor of aggregated vegetation. This simplification comes with a loss of biological detail and a smoothing out of the demographic noise engendered by stochastic individual-scale processes and heterogeneities, which is significant among others when studying the viability of small populations facing challenging fluctuating environmental conditions. This consideration has motivated the development of precise plant-centered models. The accuracy gained in the representation of plant biology has then, however, often been balanced by the disappearance in models of important plant-soil interactions (esp. water dynamics) due to the inability of most individual-based frameworks to simulate complex continuous processes. In this study, we used a hybrid modeling approach, namely integrated System Dynamics (SD)—Individual-based (IB), to illustrate the importance of individual plant dynamics to explain spatial self-organization of vegetation in arid environments. We analyzed the behavior of this model under different parameter sets either related to individual plant properties (such as seed dispersal distance and reproductive age) or the environment (such as intensity and yearly distribution of precipitation events). While the results of this work confirmed the prevailing theory on vegetation patterning, they also revealed the importance therein of plant-level processes that cannot be rendered by reaction-diffusion models. Initial spatial distribution of plants, reproductive age, and average seed dispersal distance, by impacting patch size and vegetation aggregation, affected pattern formation and population survival under climatic variations. Besides, changes in precipitation regime altered the demographic structure and spatial organization of vegetation patches by affecting plants differentially depending on their age and biomass. Water availability influenced non-linearly total biomass density. Remarkably, lower precipitation resulted in lower mean plant age yet higher mean individual biomass. Moreover, seasonal variations in rainfall greater than a threshold (here, ±0.45 mm from the 1.3 mm baseline) decreased mean total biomass and generated limit cycles, which, in the case of large variations, were preceded by chaotic demographic and spatial behavior. In some cases, peculiar spatial patterns (e.g., rings) were also engendered. On a technical note, the shortcomings of the present model and the benefit of hybrid modeling for virtual investigations in plant science are discussed.

## Introduction

### Vegetation and plant models

The modeling of vegetation dynamics has been an active topic of research since the early days of computerized ecological modeling and it has been undertaken using various techniques. Markov models have been applied to the simulation of transitions between vegetation states (e.g., Horn et al., 1975 for forest trees; Gimingham et al., 1981 for heathland; Rego et al., 1993 for mediterranean garrigue). However, as frequently pointed out, vegetation growth is not Markovian (Usher, 1992), because past and future states are dependent. As a consequence, the logical evolution leads to more precise partial differential equations models in which the dynamics of vegetation and its environment could be rendered in an aggregated manner (Cartenì et al., 2012). In these models, vegetation is considered as a whole entity and the existence of the elementary organisms composing it, namely plants, is ignored (Mazzoleni et al., 2013). In contrast, the modern development of individual-based modeling gave birth in parallel to a second approach of vegetation modeling considering population-scale behavior as the sole product of interactions between single plants. This reductionism engendered plant-centered concepts often explaining vegetation dynamics only as simplified direct competition between plants, which can be aggregated in functional relationships calibrated by regression. The ideas of fixed radius neighborhood (FRN) (Pacala and Silander, 1985; Pacala, 1986, 1987), zone-of-influence (ZOI) (Ford and Diggle, 1981; Weiner, 1982; Wyszomirski, 1983; Czárán, 1984; Hara, 1988; Wyszomirski et al., 1999; Weiner et al., 2001; Lin et al., 2014), and field of neighborhood (FON) (Berger and Hildenbrandt, 2000; Grimm and Berger, 2003) are some examples of this long-lasting tendency of compaction of ecological processes into direct plant-to-plant interactions. Aside from some notable exceptions such as in FORMIND (Köhler and Huth, 1998), mechanisms of competition for resource (e.g., root growth for groundwater uptake, rainfall interception, increase of leaf height to enhance sunlight exposure, etc) are most often not modeled explicitly. If the ZOIs of two plants overlap, they are assumed to compete for resource, and this is simply rendered by a reduction of their growth.

On the other hand, an extensive body of work, carried out with different modeling approaches, has focused on the relation between climate and vegetation. Several authors analyzed the influence of environmental factors such as precipitation, air temperature, soil water content, and wildfire on the emergence of vegetation patterns and their structure, particularly in semi-arid grazing systems (for a review, see Tietjen and Jeltsch, 2007).

As a result of the large availability of sometimes diverging approaches, the modeler has been generally facing a dilemma: either model at the vegetation level and integrate realistic hydrologic processes, or reach finer plant-scale simulations but to the price of sacrificing precision in the modeling of indirect competition mechanisms.

Some models have tried to tackle this problem, but to the price of some simplifications. The coupled vegetation-grazing model by Paruelo et al. (2008), although very useful, did not reproduce the colonization mechanisms of tussocks. It approximated dispersal as spatially homogenous (i.e., it occurred randomly across the plot) and directed (i.e., only empty cells with given conditions were targeted) (cf. 2.2.3 in Paruelo et al., 2008). Moreover, it simplified water dynamics in many aspects. For instance, soil water content was reinitialized every year, there was no runoff/runon, only simplified water diffusion and only between parcels covered by tussocks and their direct neighbors, no infiltration gain due to vegetation cover, etc. EcoHyD (Tietjen et al., 2010; Lohmann et al., 2012) on the other hand is one of the most integrated undertakings in this aspect and features realistic hydrology in several soil layers within an IBM model. Yet, aggregated vegetation is represented instead of individual plants therein.

### Modeling vegetation patterns

Intriguing vegetation patterns have been observed in different regions of the world (Clos-Arceduc, 1956; Tongway and Ludwig, 1990; Rietkerk and Van De Koppel, 1997). This phenomenon has motivated many research efforts to unveil the mechanisms responsible for this spatial organization. Much field work relying on aerial field observations, remote sensing and post-processing in GIS have been taken on. These have been able to collect patch size statistics and discover that these were following a power law distribution, meaning that the probability of finding a vegetation patch of size *n* is *n*^−β^ (with *n* > 1) (Manor and Shnerb, 2008). Further experiments gave estimations of the exponent, which proved variable between regions (Kéfi et al., 2007; Scanlon et al., 2007). In spite of these valuable results, no field experiment could explain the underlying mechanisms, meaning, and significance of this phenomenon. Confronted to the behavior of a highly dynamic system, theoretical modeling has been used to study this problem and has been fruitful in building explanative theories. The representation of such complex systems in computer models requires considerable simplification. Each model was constructed based on a restricted set of aspects and processes judged useful to try to reproduce vegetation patterns while ignoring other potential facets. Lejeune and Lefever (Lefever and Lejeune, 1997; Lefever et al., 2000; Lejeune et al., 2004) and more recently Barbier et al. (2008) proposed to model this system in terms of simplified direct facilitation-competition at the individual level. Scanlon et al. (2007) in parallel derived this system through a cellular automaton expressing probabilistic transitions depending on the biomass in the neighboring cells. Even if this proved successful, it eclipsed ecological processes by reducing the phenomenon to a matter of relative strength between birth and death rates.

By the mid-90s, several cellular automata models were formulated to simulate the formation of banded vegetation structures driven by the relations between available water and plants (Thiéry et al., 1995; Dunkerley, 1997, and for a review see Mauchamp et al., 2001). Following the use this discrete approach, several models were later presented based on an IBM paradigm, simulating vegetation dynamics and structure as well as pattern formation in different ecosystems (see Wiegand et al., 1995; Peters, 2002; Cipriotti et al., 2012, 2014).

In contrast, the problem was tackled at the vegetation scale by aggregated partial differential equations (PDE) models coupling biomass and soil moisture dynamics (Klausmeier, 1999; Von Hardenberg et al., 2001). Following the latter trend, Rietkerk et al. (2002) introduced an extension of the model by adding surface water transport and were the first to allow for an accurate study of the phenomenon by reproducing realistic surface patterns. This modeling endeavor engendered critical discoveries, namely that patterned vegetation represent bistable systems that may undergo catastrophic ecosystem shifts (Rietkerk et al., 2004) and may represent early warning signals of critical vegetation transitions (Scheffer et al., 2009). Although the relative simplicity of the model formulation allowed gaining very interesting insights on the properties of such systems, one of its major limitations is the use of constant precipitation regimes that are especially unrealistic in semi-arid environments. Moreover, the representation of plant dispersal by diffusion driven only by concentration gradients of biomass does not allow the simulation of the effects of precipitation seasonality and droughts since biomass diffusion would continue even in conditions of prolonged absence of available water.

All in all, several models featuring different mechanisms have provided possible explanations for vegetation spatial patterns. Nonetheless, without trying to integrate all these processes together in a single model, it will be difficult to falsify one or the other theory. The difference between the level of representation of vegetation—aggregated in Rietkerk et al. (2002) and individual-based in Lefever and Lejeune (1997)—also makes confrontations difficult.

In this paper we present a new hybrid model that integrates the System Dynamics (SD) and Individual Based (IB) modeling approaches. This work intends to illustrate the importance of the dynamics of single plants to explain the spatial self-organization of vegetation. To do so, we analyze the model behavior in relation to plant-specific parameters (seed dispersal distance and reproductive age) and climatic inputs (precipitation intensity and seasonality). In fact, dispersal range has been shown to influence the outcome of a spatially explicit population model (Johst and Schöps, 2003), and modeling applications have been investigating how both patch distribution in the physical landscape and plant dispersal capability can generate demographic noise that affects the dynamics of vegetation (Higgins and Cain, 2002; Realpe-Gomez et al., 2013; Sheffer et al., 2013). Therefore, the importance of the representation of biological dispersal is also evaluated in this study through a comparison with previous reaction-diffusion models.

## Methods

### Description of the hybrid plant population model

For the sake of standardization, we follow hereafter the 7-points Overview—Design concepts—Details (ODD) model description protocol formulated by Grimm et al. (2006, 2010).

#### Purpose

The purpose of this model here is to better understand the mechanisms of spatial pattern formation. It was designed to reproduce vegetation-soil interactions with water transport and individual plants in a spatially-explicit environment.

#### Entities, state variables, and scales

**Figure 1:**
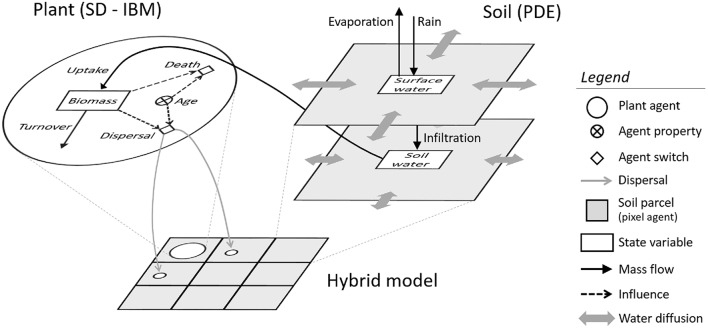
**Schematic view of the model structure and featured processes**. The hybrid model simulates plant agents (modeled with a combined Individual-Based and System Dynamics approach) and surface and soil water dynamics (modeled with System Dynamics and Partial Differential Equations). The combination of SD and IBM components follows a joint use of reference case 2a and 3a (Vincenot et al., 2011).

This model includes two entities: plants and soil parcels (Figure 1). Soil properties are described by two state variables keeping track of the volume of water flowing on the surface and infiltrated in the soil. Water in both layers is diffused between adjacent soil parcels. While water enters the system through rainfall, it is released through evaporation and consumption by plants. The latter are entities represented individually in an open-ended space and defined mainly by their age and their biomass (itself dependent on a growth and a senescence process). The life cycle of plants is represented in a basic manner in the model. Depending on their biomass, plants can disperse seeds to produce offsprings. Finally, when some critical conditions are met (i.e., advanced age or low biomass), plants can die.

Plant individuals are this way not aggregated and are instead modeled explicitly within parcels. Both plants and soil parcels have bidimensional spatial coordinates positioning them in two superimposed square spaces (continuous and discrete respectively). Plot dimensions are set based on number of soil parcels and their side length (e.g., the 400 × 400 m plot is simulated as 100 × 100 parcels, each covering 4 × 4 m). Simulations are run with a time step of 0.2 day (i.e., ca. 5 h) and render the evolution of the vegetation biomass over years.

#### Process overview and scheduling

Processes take place in the following order inside of the model: plant metabolism (water absorption, biomass growth, and senescence), seed dispersal, water dynamics in the soil (precipitation, infiltration, evaporation, and finally water diffusion). The global model runs in hybrid time with plant metabolism and water dynamics calculated in continuous time, and seed dispersal and plant death happening as discrete asynchronous events. Note that for continuous computations, the classical Runge-Kutta 4 (RK4) method with an integration step (Δt) of 0.001 day was used to compute the results reported in this study.

#### Design concepts

##### Basic principles

This study follows conceptually the work on vegetation modeling by Realpe-Gomez et al. (2013). More precisely, it aims to merge two concurrent modeling views and tactics. A first class of models (e.g., Peters, 2002; Barbier et al., 2008 and following works) could render vegetation patterns with an individual-based approach simulating direct simplified competitions between plants, while other projects could explain this phenomenon on the basis of results from an aggregated approach featuring vegetation-soil interactions expressed in the form of partial-differential equations (PDE) (e.g., Rietkerk et al., 2002 and similar). The will to fuse both approaches—an individual-based representation of plants with plant-soil interactions—motivates the creation of the model presented here. The individual-based dimension is exploited here only for metabolism and dispersal, while direct inter-individual competition, although attainable within this framework, is not implemented.

Also, another fundamental principle that underlies this work is the compliance with the temporal and spatial nature of the processes featured. Particularly, plant metabolism and water dynamics are computed in continuous time, while plant dispersal and plant death take place as stochastic and discrete events. Besides, an improved accuracy in the representation of the spatial process of plant dispersal is sought. Instead of a physical diffusion of vegetation, seed dispersal is explicitly modeled here.

For these matters, the implementation relies on the System Dynamics (SD)—Individual-Based (IB) hybrid modeling framework (Vincenot et al., 2011) offering the flexibility necessary to realize the integration of these different aspects inside of an individual-centered vegetation growth model and allow for the concurrent use of discrete and continuous time computation engines. This conceptual framework aims to facilitate the accurate and understandable design of models in ecology by relieving the modeling process from adverse technical considerations (Vincenot and Moriya, 2011; Vincenot et al., 2015).

*Emergence:* The demography of the plant population as well as its spatial distribution are obviously emergent properties of the interaction between plant individuals through indirect competition for groundwater. As a result, soil water content is also emerging from the same interplays.

*Interaction:* Soil parcels interact directly by exchanging surface water and groundwater. This process follows the basic principle of water diffusion. In a different manner, plants also interact, but indirectly only through the competition for a mediating resource (sc. water).

*Stochasticity:* Stochasticity is integrated in various processes included in the simulation of plants. First, at initialization, plant biomass are set randomly to avoid synchronicity. Furthermore, death is stochastic. When the biomass of a plant falls under a death threshold, the plant may die each day with a probability equal to *p* = 1−*Biomass*∕*deathThreshold*. Also, while the amount of seeds dispersed is simply proportional to the plant's biomass for large plants, for low biomasses engendering an average of less than 1 seed/timestep, it is determined probabilistically depending on the plant's biomass. The lower the biomass, the lower the chance to disperse a seed. Ultimately, the location of seedlings is also chosen randomly following a uniform direction from the parent plant (sc. isotropic dispersal) and a distance drawn from a lognormal dispersal kernel [cf. Section Plant Life Cycle and Seed Dispersal (IB)].

*Observation:* All the different state variables are considered in the following experiments. However, the most reported observation concerns the total vegetation biomass and its spatial distribution.

#### Initialization

When a simulation starts, a grid of soil parcels, the number of which depends on the resolution chosen, is created to cover the bidimensional space. State variables representing surface water and soil water are initialized with values for bare soil conditions, that is to say *R*∕(α*W*_0_) and *R*∕*r*_*w*_, respectively. Plants are then instantiated with an initial biomass between 1 and 2 g and an age drawn uniformly in the range 1 to *l* to avoid artificial synchronicity within the population. The location and number of plants depends on the experiment undertaken. In some cases, plants are grouped in patches covering 1% of space (i.e., “aggregated” initial distribution), while in others they are spread randomly. Other exogenous parameter values used in the hybrid model and their sources can be found in Table 1.

**Table 1:** **Default parameter values used in the hybrid model**.

**Aspects**	**Parameter**	**Symbol**	**Value**
Plant Metabolism	Biomass	*B*	1 g (initial value)
	Water to biomass conversion rate	*b*	10 g·mm^−1^·m^2^
	Maximum water uptake	*g*_*max*_	0.05 mm^−1^·m^2^·g^−1^·d^−1^
	Half-saturation constant of plant growth and water uptake	*k*_1_	3 mm^−1^·m^2^
	Plant size limiting factor	*K_*p*_*	800 g^†^
	Biomass s rate	*d*	0.3 d^−1^
Plant life cycle and dispersal	Age	*A*	[1, *l*] (initial value)
	Mean life expectancy	*l*	365 d
	Death threshold (based on biomass ratio)	*d_*b*_*	0.8
	Reproductive maturity	*m*	0 d
	Biomass-to-seedlings factor	*s*	0.002 seeds ·g^−1^·d^−1^
	Mean dispersal distance (dispersal kernel)	μ	10 m
	Shape (dispersal kernel)	σ	1.5
	Maximal plant density (for seedling establishment)	*K_*a*_*	30 g·m^−2^
Soil hydrology	Surface water	*O*	$\frac{R}{\alpha {\text{W}}_{0}}{\text{mm}}^{-1}\cdot {\text{m}}^{2}$ (initial value)
	Groundwater	*W*	$\frac{R}{{\text{r}}_{\text{w}}}{\text{mm}}^{-1}\cdot {\text{m}}^{2}$ (initial value)
	Precipitation	*R*	0.3–2.1 *mm*^‡^
	Maximum infiltration rate	α	0.1 *g*^−1^·d^−1^
	Infiltration saturation constant	*k*_2_	5 g
	Infiltration rate on bare soil	*W*_0_	0.15
	Evaporation and drainage rate	*r*_*w*_	0.1 d^−1^
Water diffusion	Surface water diffusion coefficient	*D*_*O*_	10 m^2^·d^−1^
	Groundwater diffusion coefficient	*D*_*W*_	0.01 m^2^·d^−1^
	Parcel side length	Δ*x* and Δ*y*	4 m
Simulation engine	Delta time (DT) (time steps)	Δ*t*	0.2 d

*When not specified otherwise, the parameters are dimensionless quantities. The SD equations were solved by the Runge-Kutta (RK4) integration method. Parameters of the soil hydrology and water diffusion submodels values were kept in the range within which Rietkerk et al. (2002) performed sensitivity analysis. The dispersal kernel is inspired of Greene et al. (2004) and Quinn et al. (2011), the death threshold of Hacker et al. (2006), while the metabolic submodel was parametered using data from the literature and “best guesses” (mainly from Rietkerk and Van De Koppel, 1997)*.

† *Theoretical limit balanced by growth and biomass turnover variability. Set to have a maximal effective plant biomass of ~120 g*.

‡ *Precipitation is reported here in millimeters due to the classic measurement system. Nevertheless, strictly speaking, the dimension should be mm^−1^·m^2^·d^−1^ for unit testing*.

#### Input data

No external input data are used in this study.

#### Submodels

The hybrid model can reproduce the dynamics of plant-soil interaction by uniting a spatial water dynamics submodel (mainly in SD, but with spatial localization and diffusion processes coded in IB modeling), a plant metabolism submodel (in SD), and a plant life cycle and dispersal submodel (in IB modeling) (Figure 1). The combination of SD and IB components follows a joint use of reference case 2a (“Independent dynamic individuals with fixed spatial location”) to model plant metabolism and reference case 3a (“One-to-one interaction between individuals and a space of fixed SD submodels”) to render the interaction between plants and soil hydrology as identified in Vincenot et al. (2011). The description of these submodels is given hereafter, while the values of parameters are given in Table 1.

##### Water infiltration (SD) and diffusion (IB)

This submodel is based on an adaptation of the equations by HilleRisLambers et al. (2001) and Rietkerk et al. (2002). When rain falls (i.e., the precipitation process symbolized by *R*), it forms water on the surface of the soil (*O*). Part of it flows to other parcels (i.e., the diffusion process, dependent on a surface water diffusion coefficient *D*_*O*_), while a fraction of it follows an infiltration process depending on properties of the soil (the maximum infiltration rate α, infiltration saturation constant *k*_2_, and infiltration rate on bare soil *W*_0_) and on the vegetation biomass ($\underset{i}{\sum }B_{i}$, with *i* the index of the plant in the population), which facilitates the percolation process (Rietkerk and van de Koppel, 2008). Once in the soil, groundwater can leave the system through evaporation and drainage (depending on the relevant rate r_w_), diffusion to neighboring soil (relatively to a soil water diffusion coefficient *D*_*W*_), or by being drawn by the overlying vegetation. The diffusion process takes place in the form of a transfer of water between individuals incarnating soil parcels. Mathematically, simple diffusion is achieved by the solving of a single Laplacian diffusion term (*D*_*w*_·△*W* and *D*_*O*_·△*O*) as visible in Equation (1). This is justified by hydrological mechanisms involving a gradient of pressure between low and high water densities (Bear and Verruijt, 1987; HilleRisLambers et al., 2001).

(1) $$\begin{array}{l} \frac{\partial W}{\partial t}=\alpha \cdot O\cdot \frac{\sum_{i}B_{i}+k_{2}\cdot W_{0}}{W+\;k_{2}}-g_{max}\cdot \frac{W}{W\;+\;k_{1}}\cdot \sum_{i}B_{i}\\ \;-\;r_{w}\cdot W+\;D_{w}\cdot \Delta W\\ \frac{\partial O}{\partial t}=R-\alpha \cdot O\cdot \frac{\sum_{i}B_{i}+k_{2}\cdot W_{0}}{W+\;k_{2}}+D_{O}\cdot \Delta O \end{array}$$

Equation (1): Water dynamics and diffusion in the soil.

The non-linear system summarized by the set of differential Equation (1) was translated into System Dynamics to allow for a better visualization and an easier analysis of the system (Datasheet S1.1). In SD terminology, each state variable is symbolized by a *stock*, while the processes moving food or energy between them correspond to *flows* linking together the previous stocks.

##### Plant metabolism (SD)

Plant metabolism is integrated in the model in the form of a set of simple processes. Basically, the only state variable—the plant's biomass—is decreased by a natural turnover rate *d*, while it is increased by anabolic processes dependent on water uptake and a water-to-biomass conversion rate *b*. Water uptake is a function of plant biomass *B*_*i*_ (with *i* the index of the plant in the population), soil water volume *W*, a half-saturation constant of plant growth and water uptake *k*_1_, and a maximum water uptake *g*_*max*_. The plant's biomass is also limited by a limiting factor *K*_*p*_, here set so that one plant cannot grow over ca. 120 g (Equation 2).

(2) $$\frac{dB_{i}}{dt}=B_{i}\cdot b\cdot g_{max}\cdot (1-\frac{B_{i}}{K_{p}})\cdot \frac{W}{W\;+\;k_{1}}-B_{i}\cdot d$$

Equation (2): Plant metabolism.

Aside from metabolic calculations, this submodel also computes number of seeds to disperse. This is computed based on the plant's biomass *P*(*disperse*) = *B*_*i*_·s·*l*∕*m*·Δ*t*, with s being a biomass-to-seedlings factor, l the mean plant lifespan, and m the age of reproductive maturity. If *P(disperse)* is superior to one, it represents the numbers of seeds to disperse, otherwise the probability to disperse a seed. As for the previous submodel, all the equations were implemented in SD and were computed in continuous time.

##### Plant life cycle and seed dispersal (IB)

Plants are autonomous entities possessing individuality. One characteristic of these individuals is their coordinates in a spatial environment in which water availability varies depending on location, and another is their age, which is automatically incremented at each timestep.

The life cycle of plants depends on these two fundamental properties. In the submodel, when a seedling takes root, its initial biomass is set to 1 g and its age to 0 day. When it reaches maturity (*m*), the plant starts reproducing. During its life, it can die from catabiosis as explained in Section Design Concepts (“stochasticity”). Ahead of this programmed quietus, biomass decay is provoked by water stress. When the relative decrease in plant biomass (*B*_*i*_/*Bmax*_*i*_, with *Bmax*_*i*_ representing the largest biomass reached by the plant so far) falls under the threshold value *d*_*b*_, a stochastic death process comes into effect (sc. death happens with a probability $1-\frac{B_{i}}{Bmax_{i}\cdot d_{b}}$).

The active part of the plant's life deals with metabolism [cf. Section Plant Metabolism (SD)] and biological dispersal. The latter is modeled in the present submodel in form of seed dispersal. More precisely, following the results of Higgins et al. (2003), seedling dispersal is represented here instead of propagule dispersal. Seed loss (due to factors other than water stress or random mortality; e.g., predation) is in part implicit. Based on the production of seeds computed by the metabolic submodel, the number and spatial location of seedlings to disperse is calculated. For this purpose, a dispersal kernel was considered as the best option. The literature has shown that the empirical determination of the dispersal range is very difficult to obtain due to the intersection of seed shadows as well as the trickiness of field work methods (Clark et al., 1999). Incidentally, models have been a major source of design and validation for dispersal kernels. Here, we chose to rely on a lognormal distribution *lnN*(μ, σ^2^), which proved to be the most fitting solution and the only one validated on experimental data instead of simulations (Greene and Johnson, 1989; Greene et al., 2004; Quinn et al., 2011). Due to the lack of information about species subject to vegetation patterning, the dispersal kernel could only be fitted approximately based on an anterior study on grass species of the *Miscanthus* genus (Quinn et al., 2011). Also, the design of the shape of the distribution was based on a biological consideration. Several studies have clearly shown that antitelechory and atelechory are common among taxa living in arid regions (Ellner and Shmida, 1981), which means that adaptation for long-range dispersal is probably rare among vegetation subject to water stress and self-organization as studied here. The dispersal kernel was then made leptokurtic to render this. A seed is then dispersed in the submodel by selecting randomly a direction between 0 and 2π rad, and then drawing a distance using the dispersal kernel. Note that to avoid inaccurate border effects, a location attributed outside of the limits of space is ignored and the related seed is lost. Also, seedling establishment is prevented if biomass density in the area surrounding the dispersal point is greater than *K*_*a*._

##### Practical implementation and fusion of the SD and IB model components

All the submodels were implemented in XJTechnologies AnyLogic 6.8.1. This framework relies entirely on the Java object-oriented language and allowed for the SD and IB model components to be integrated as Java classes. Conceptually, the plant metabolism submodel and the life cycle and seed dispersal submodel were virtually embedded inside of the class of agent representing plants, while the water dynamics submodel was integrated in the class of soil parcels. Instances of this class were arranged at initialization in a static grid-like network to exchange water and drive thereby the hydrological diffusion process.

At several places in the submodels, so-called “hooks” allowing for data exchange between the SD and IB model components were placed. For instance, the life cycle and seed dispersal IB submodel operating in discrete time had to be able to query the values of the biomass stock and the seedlings production computed continuously in the SD metabolic submodel. Likewise, the synchronous exchange of information on water level between IB soil parcel agents necessary to compute the spatial water flows obviously required mutual interaction between the IB diffusion algorithm and SD stocks (i.e., *O* and *W*).

Water density in the soil was also queried by plant agents, which in return inform about their biomass for the calculation of biomass density. In this manner, it should be noted that plants do not really compete for water within the same parcel. They all have access to the same water density, and each plant consequently draws a volume of water proportional to its biomass.

### Simulation experiments, parameters, and analysis

Two main experiments were performed to study on one hand the relationship in this individual-based model between precipitation, pattern formation, and population structure, and, on the other hand, the consequences of temporal variations in rainfall on vegetation spatial organization. Parameters values are given in Table 1, and only departures from this default configuration are mentioned hereafter.

Experiment 1: The emergence of spatial self-organization in an individual-based paradigm was analyzed.

- The model was run across a wide range of precipitation (0.6–1.7 mm) and two alternative conditions for initial plant spatial distribution [either randomly scattered over the map, or with plants grouped in patches covering 1% of the plot as in Rietkerk et al. (2002)] to simulate a 400 × 400 m plot during 10 years. The spatial patterns obtained were compared with known field observations as well as results from previous diffusive models. Population structure in terms of age and biomass was also observed. In this case, the simulations were replicated 300 times per rainfall configuration to get statistically significant results.
- The effects on vegetation pattern formation of plant-level parameters, namely mean seed dispersal range and reproductive age, were assessed through parameter variations (in the range 1–100 m, and 0–240 days respectively, with *s* = 0.001, 70 runs for each parameter). Plants were grouped as initial condition (IC) and we checked the spatial distribution of the plant population after 5-year runs at a 200 × 200 m scale. Note that initial patch and plant number were decreased accordingly to simulate a smaller area of the plot featured in Experiment 1A. For each run, two indices of population spatial dispersion were calculated using the software package PASSaGE v2 (Rosenberg and Anderson, 2011): the Index of Patchiness (IP; Lloyd, 1967) and the Index of Cluster Size (ICS; David and Moore, 1954).

Experiment 2: The effects of variations in the precipitation regime were assessed. Two types of variations were investigated.

- A sudden shift toward drier climate was simulated. A population (250 individuals, aggregated IC) was initialized on a 200 × 200 m plot and allowed to grow under 1.3 mm precipitation during 1500 days, after which it was exposed to a rainfall regime of 0.8 mm. Mortality due to age was turned off after the shift to remove natural death. The effect of this shift on the population's spatial organization and mortality across age and biomass classes was studied in 100 replicated runs. Furthermore, as in Experiment 1B, patchiness and cluster size were measured before and after the shift. For both indices, the significance of differences between pre-shift and post-shift conditions was assessed by non-parametric Wilcoxon test for paired samples, after verifying the heteroscedasticity through Levene's test. As a side experiment, the viability of the population depending on timing (either *t* = 1 or 1500 days) or amplitude (down to –1.0 mm) of the shift and on initial condition in spatial distribution was explored in 40 replicated runs per configuration with an initial population of 2000 individuals.
- Seasonal variations in water availability were introduced in the model in the form of sinusoidal changes in precipitation, which rendered two seasons–one dry, the other rainy. Amplitudes up to 1.3 mm ± 0.9 mm were investigated in 100 runs. The impact on plant spatial organization on a 200 × 200 m plot was observed over 12 years.

## Results and interpretations

### Experiment 1: spatial self-organization

#### Patterns exhibited by the hybrid model under different precipitation regimes

##### Pattern formation

**Figure 2:**
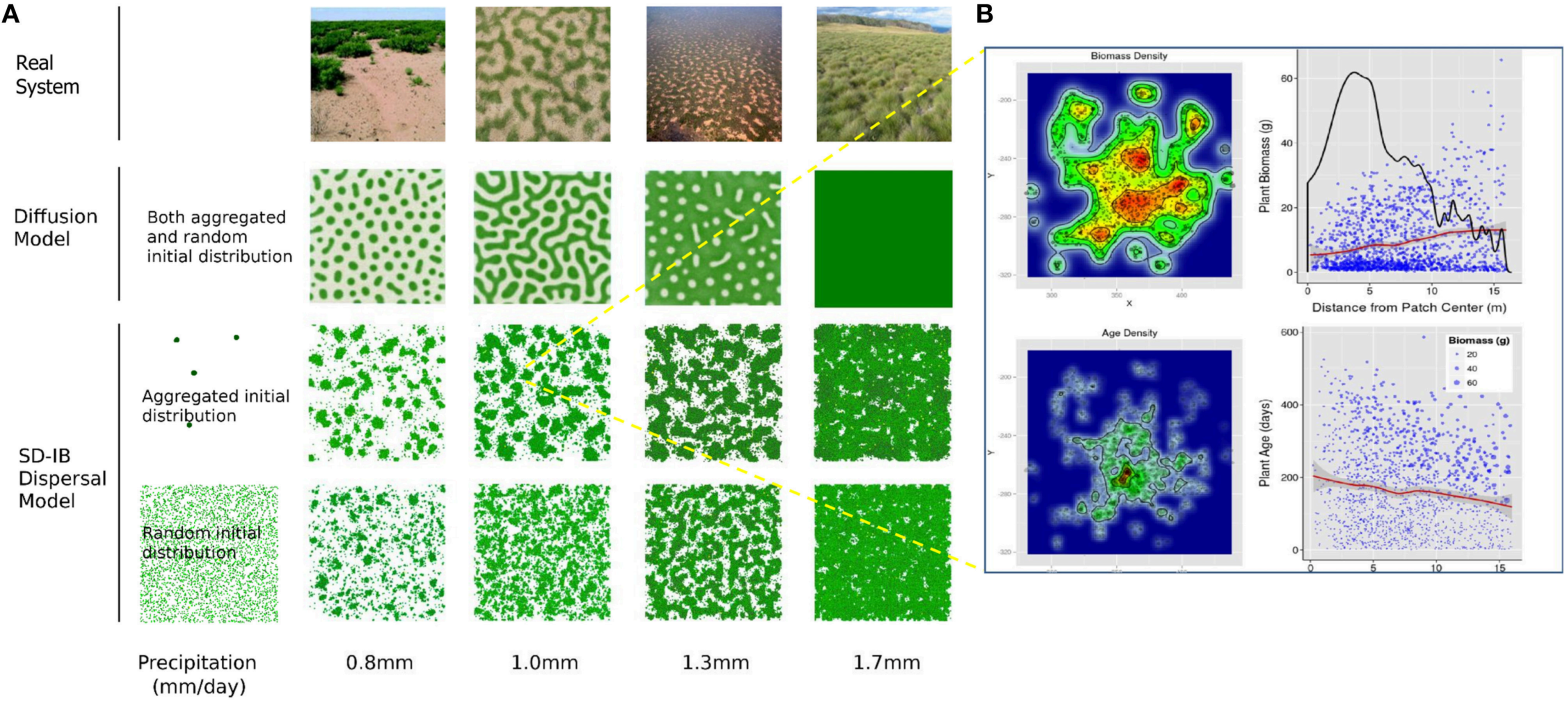
**Impact of precipitation and initial condition on pattern formation**. **(A)** Observed and simulated patterns in different precipitation regimes. The first row shows photographs of observed vegetation patterns. The second row shows results of the diffusive model by Rietkerk et al. (2002). Third and fourth rows refer to 5-year simulations of the presented hybrid SD-IB model with two different initial distribution of plants (Aggregated distribution: plants are grouped in 1% of the available space; Random distribution: plants are randomly spread over the map). The simulated areas represent a 400 × 400 m plot. Reproductive age was set to 0, *D*_*O*_ = 10, *D*_*W*_ = 0.01, *s* = 0.002, *B*_*init*_ = 1, *n* = 1000 plants. Other parameter values are reported in Table 1. See also Video 1 included as **Supplementary Material**. Photos illustrating the type of vegetation observed in the field for given precipitation ranges are included, with from left to right: (i) In New Mexico, Shrubland exhibiting spotted patterns of vegetation with medium to large corridors of bare soil (32°36'29.39”N 106°49'4.81”W) (< 200 mm annual rainfall). Photograph: Brandon T. Bestelmeyer. (ii) Labyrinth of perennial grass *Paspalum vaginatum* observed in the northern Negev (over 200 mm annual rainfall). Reprinted with permission from Von Hardenberg et al. (2001). Copyright (2001) by the American Physical Society. (iii) Aerial view of a gapped bush plateau dominated by *Combretum micranthum* and *Guiera senegalensis* in the Nigerian part of the W regional park (12° 22' 42.24” N, 2° 24' 10.8? E) (ca. 600 mm annual rainfall). Photograph by Nicolas Barbier released under the Creative Commons Attribution-Share Alike 3.0 Unported license. (iv) Subalpine vegetation in the state of Victoria, Australia (over 700 mm annual rainfall). Photograph courtesy of the Biodiversity Branch, Department of Environment, Land, Water, and Planning, State of Victoria, Australia. **(B)** Structure of a self-organized vegetation patch, with the individual-scale biomass (top half) and age (bottom half) of plants. On the left, the 2D biomass and age density is plotted in a color gradient from blue to green to red for increasing values, and with the individual-scale biomass and age represented as the radius of the points representing plants. On the right, a transect view of the patch is shown with plant biomass/age plotted against distance from the patch center, and a non-parametric local regression (LOESS) provides a smooth average of biomass/age (red line). In the case of the biomass plot (top-right), biomass density is also provided (black line), whereas in the age plot (bottom-right) individual biomass is concurrently represented in the form of point size.

A range of spatial patterns emerged when running the model with different values of precipitations (Figure 2A). These mirrored closely what could be observed in various regions of the world for vegetation suffering from different degrees of water stress (Figure 2A, upper row). When facing low precipitation, plants grouped in high-density “spots.” With lower water shortage, they formed a labyrinth pattern with corridors of bare soil exhibiting a width inversely proportional to the average rainfall. With medium precipitation, most of the surface was populated by plants, except some empty “gaps.” As predictable, when large scale competition for resource was inhibited by a widespread availability of water; the model outputted a relatively uniform coverage.

Interestingly, the patches of plants were slightly amorphous and formed clear yet disordered patterns that demonstrated some variability depending on initial conditions. When plants were spread randomly at initialization, the population looked disintegrated and composed of series of small dots or groups of differing sizes (Figure 2A, fourth row). On the opposite, with an “aggregated distribution” at initialization [i.e., plants grouped in 1% of the parcels as in Rietkerk et al. (2002)], vegetation grouped in a smaller number of larger patches of variable shapes separated by wide empty areas or corridors of bare soil (Figure 2A, third row). The differences observed between random and aggregated initial condition could be explained by the fact that the width of the patches and of the corridors was also directly influenced by the number of soil patches that were populated at initialization (see Datasheet S1.2).

On the whole, outputs from the hybrid model resembled results from diffusive models, although a few significant differences could be noted (see Section Importance of Climate Variability).

##### Patch spatial structure

Unlike diffusive models, the hybrid model produced irregular patches, the biomass density of which was decreasing with distance from the patch center but which was spatially distributed over several hotspots of high biomass density (Figure 2B, top). The average individual biomass was, however, increasing with distance from the center (Figure 2B, top-right, red line), with large plants on the periphery of the patch (Figure 2B, top-right) and of hotspots (Figure 2B, top-left).

Spatially, older plants were present within the core of the patch, with generally one area of particularly high density of old plants in the center of the patch (Figure 2B, bottom-left). The average age of plants proved to be decreasing with distance from patch center (Figure 2B, bottom-right, red line). Unlike the foregoing trends in biomass, which were lasting, these proved to be fading with time, with the average plant age curve becoming progressively flat with consecutive dispersal events. It is worth noting that individual biomass proved to be limited by the plant's age but also concurrently by proximity to the patch center (Figure 2B, bottom-right, see point size).

##### Effect on population age and biomass distribution

**Figure 3:**
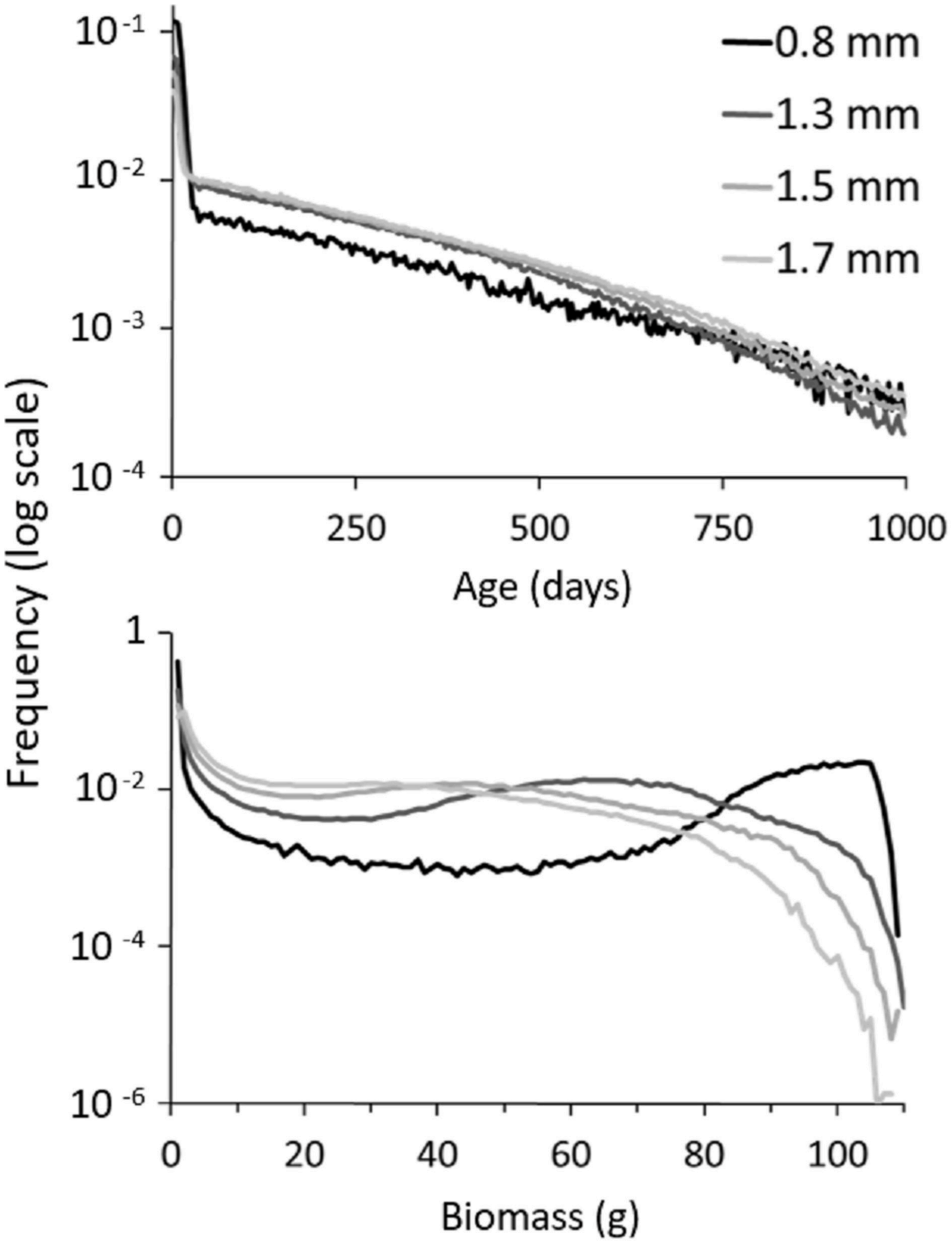
**Relative frequency of age and biomass under different precipitation regimes (0.9, 1.3, 1.5, and 1.7 mm)**. At initialization, 250 plants were grouped in 25 patches on the 200 × 200 m plot. The experiment was run over 5 years and replicated 300 times for each rainfall configuration. (*s* = 0.001; other parameters, refer to Table 1).

Higher water availability increased non-linearly the biomass density supported by the plot (i.e., 0.4, 6.5, 10.3, and 13.8 g/m^2^ at 0.9, 1.3, 1.5, and 1.7 mm respectively). The effect of precipitation was also visible in the age and biomass structure of the population (Figure 3). The drier the climate, the lower the mean age of the population (e.g., 200 vs. 228 g for 0.9 vs. 1.3 mm rainfall; mean confidence ±1.7 and ±0.6 g respectively) and the higher the prevalence of young plants (less than a month old), which ranged from 15% of the population at 1.7 mm to 48% at 0.9 mm.

On the contrary, plants were on average significantly larger the drier the climate, with mean plant biomass increasing from 24.4 g at 1.7 mm to 26.3, 31.2, and 45.4 g (mean conf. < 0.3) at 1.5, 1.3, and 0.9 mm, respectively. The frequency distribution of plant biomass was bimodal. The first peak, representing seedlings, was fixed around 1 g and inversely proportional in height to precipitation (e.g., 0.42 at 0.9 mm vs. 0.08 at 1.7 mm). The second peak position varied. It shifted rightwards and also increased in amplitude with decreasing precipitation. For instance, under 1.7 mm rainfall, it was barely noticeable at 30 g with a frequency of only 0.012 for the peak summit (i.e., the 30-31 g category), whereas under 0.9 mm rainfall, it culminated at 103 g and represented a frequency twice as important (i.e., 0.2 for the 103-104 g category). Under water stress, plants were therefore on the whole conspicuously larger albeit scarcer (e.g., 0.009 vs. 0.57 plant/m^2^ at 0.9 and 1.7 mm, respectively).

One explanation for this phenomenon may be that precipitation is positively correlated with increase in total biomass and mean patch size. The former is obvious, while the latter comes from the biomass front progressing further outwards when more water is available in the periphery of the patch. As a consequence, the increase in total biomass generates an increase in number of seeds dispersed, which increases seedling density. The increase in patch size on the other hand also contributes in increasing within-patch dispersal. As a consequence of these processes, mean individual biomass decreases (see Datasheet S1.3 for a detailed explanation).

#### Influence of plant-scale dynamics on pattern formation

**Figure 4:**
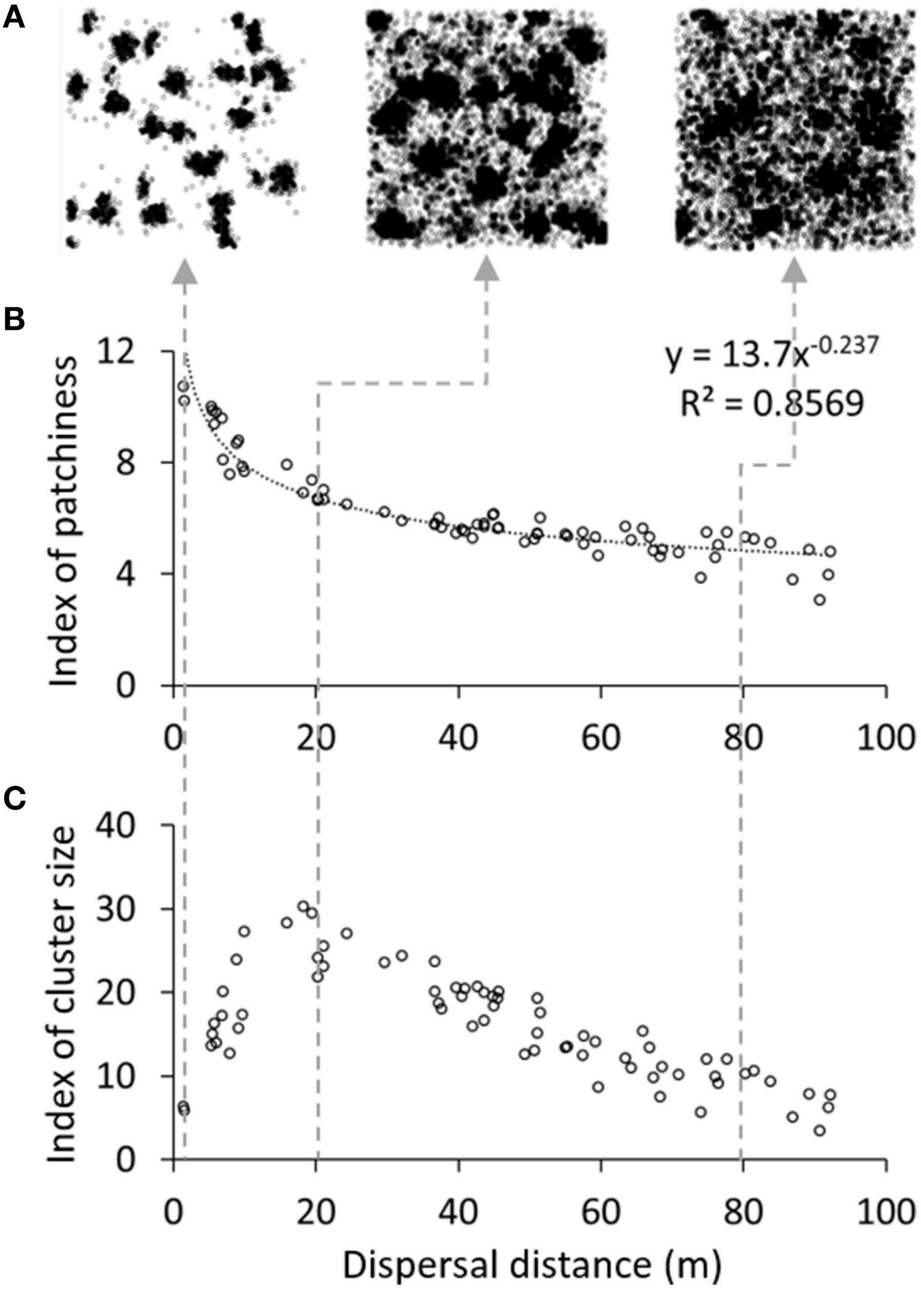
**Influence of dispersal distance on vegetation patterns**. The top panel **(A)** illustrates typical spatial distributions at the end of simulations with 1, 20, and 80 m mean dispersal distance (from left to right). Each plot represents the effect of dispersal distance on **(B)** the index of patchiness and **(C)** the index of cluster size. At initialization, 250 plants were grouped in 25 patches on a 200 × 200 m plot and the experiment was run over 5 years (Default parameter values).

The appearance of spatial patterns was influenced by both dispersal distance and reproductive age. Increasing the dispersal distance globally tended to scatter plants (Figure 4A). While this phenomenon was established quantitatively through a continuous decrease of the IP over the whole parameter range (Figure 4B), the ICS indicated that absolute clumping actually increased until dispersal values of ca. 20 m, after which it declined (Figure 4C). An intuitive explanation for this observation is that, in the lower range, increased dispersal distance enables plants to colonize neighboring areas and thereby enlarge patches, whereas too long dispersal distances do not benefit the source patches anymore, resulting on the contrary in a disaggregation of patches. Increasing reproductive age, on the other hand, resulted in a monotonic spatial fragmentation of the population (see ICS in Datasheet S1.4).

### Experiment 2: resistance to climatic changes

#### Precipitation shift

**Figure 5:**
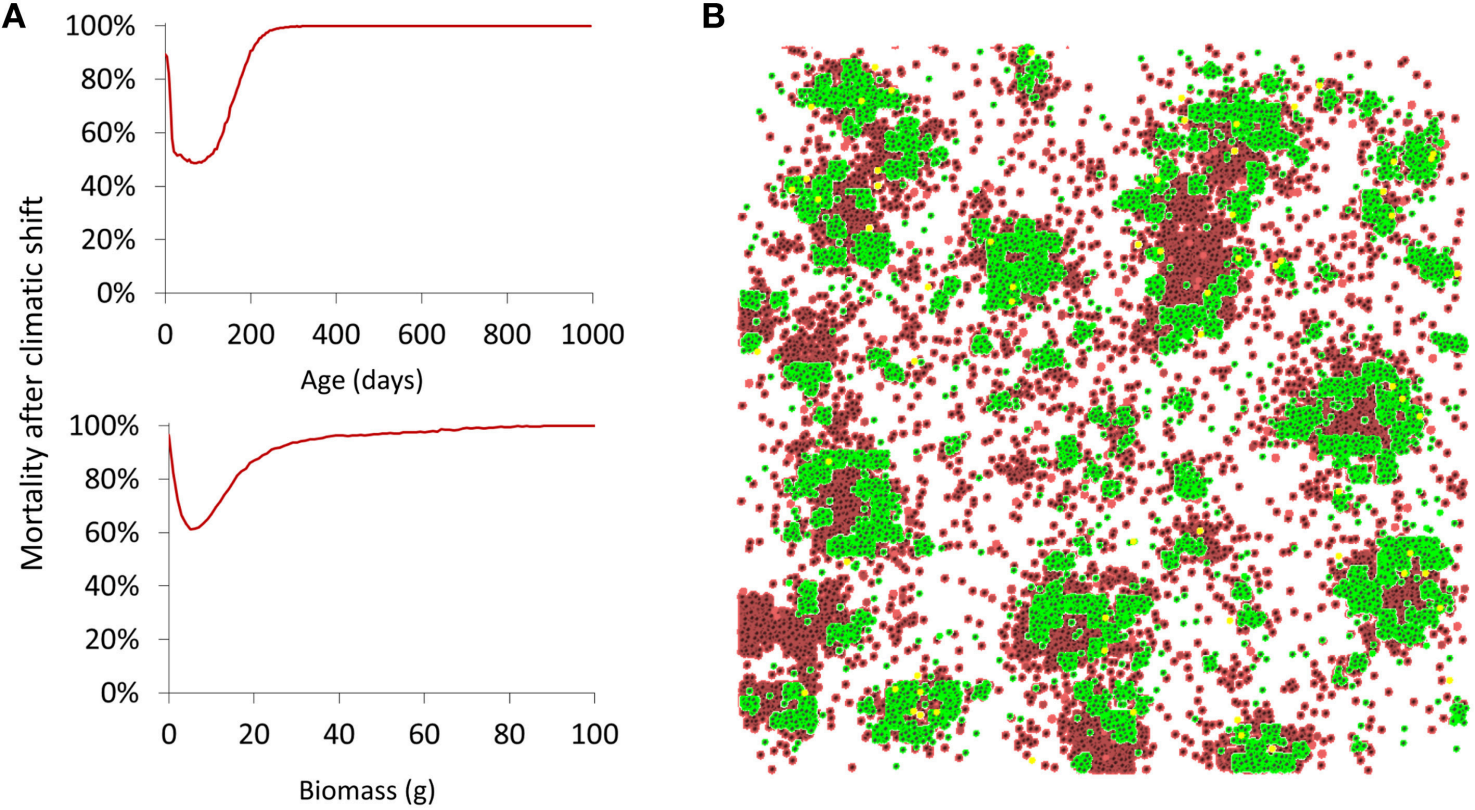
**Effect of a shift in precipitation regime (1.3 –> 0.8 mm) on vegetation**. **(A)** Plant mortality depending on age and biomass averaged over 100 replicated runs. **(B)** Example of a simulated plot (200 × 200 m) right after the precipitation regime shift. The plot shows surviving individuals (green) and the death (red) of old individuals in the central areas of the patches and young isolated individuals (Default parameter values).

Effects of a shift from 1.3 to 0.8 mm rainfall were visible only about 1 month after its occurrence during which soil water levels could buffer to some extent the reduction in precipitation. The post-shift mortality of plants (Figure 5A) revealed that surviving plants were mainly individuals between 1 week and 1.5 months old, and individuals with a biomass between 5 and 15 g before the shift. Visually, this resulted in seedlings—isolated or on patch edges—as well as plants located in small patches or in the core area of larger patches to disappear (Figure 5B). The resulting spatial distribution of plants then remained, as surviving plants were mostly not able to recolonize the lost ground.

Compared to pre-shift conditions, population spatial aggregation significantly changed in both patchiness and cluster size. The simulated shift broke down existing patches, as visible through a decrease in ICS (from 16.04 ± 6.10 to 10.89 ± 0.64, *Z* = 3.51, *p* = 0.00045), yet reduced the scattering of plants (i.e., by removing fragile patches), as rendered by a significant albeit slight increase in IP (from 7.07 ± 1.49 to 8.11 ± 0.45, *Z* = 3.40, *p* = 0.00066, Wilcoxon test for paired samples). Interestingly, for both patchiness and cluster size, the variability among simulation runs was significantly higher in pre-shift conditions than after the shift [IP: *F*_(1, 58)_= 45.7, *p* = 7.4 × 10^−9^, ICS:*F*_(1, 58)_= 70.9, *p* = 1.2 × 10^−11^, Levene's tests for homogeneity of variances], indicating that stochastic effects on spatial patterns are less pronounced under higher climatic severity.

Note that it could be argued that, in the particular case of plants less than 1 g or less than a week old, it would not be possible here to discern shift-related death from natural death (due to the abundant dispersal of doomed seedlings). To address this concern, we confirmed through a comparison with additional runs without shift that even in this subpopulation post-shift mortality was still higher than usual seedling death (0.97 vs. 0.77 for the 0–1 g biomass class, and 0.77 vs. 0.52 for the 1–7 days age class).

While it was not the main focus of this experiment, shifts of different intensities (up to 1.0 mm negative change) and timing were also tested to determine under different configurations the lowest viable precipitation. The spatial distribution at initialization (random or aggregated IC) influenced the population structure before the shift, with random distribution at initialization favoring a higher proportion of seedlings and higher plant biomass. Random IC thereby decreased population viability (i.e., its chances of survival in repeated simulations) compared to aggregated IC. For instance, under the same parameter set as in this experiment, a population initialized with random IC had one chance out of three to go extinct after a −0.78 mm shift (and >90% chance after a shift of 0.82 mm), while under aggregated IC, it always survived down to −0.9 mm. An earlier timing of the shift exacerbated these differences. Globally, if the shift was to occur at the beginning of the simulation (*t* = 1), the viability of the population significantly decreased whatever the IC (e.g., for aggregated IC, die outs then happened from −0.82 mm), indicating the beneficial effect of vegetation self-organization into patterns in this aspect. The negative effect was most important in the case of random IC though, for which shifts of only −0.3 mm already triggered extinctions.

#### Seasonal variations

**Figure 6:**
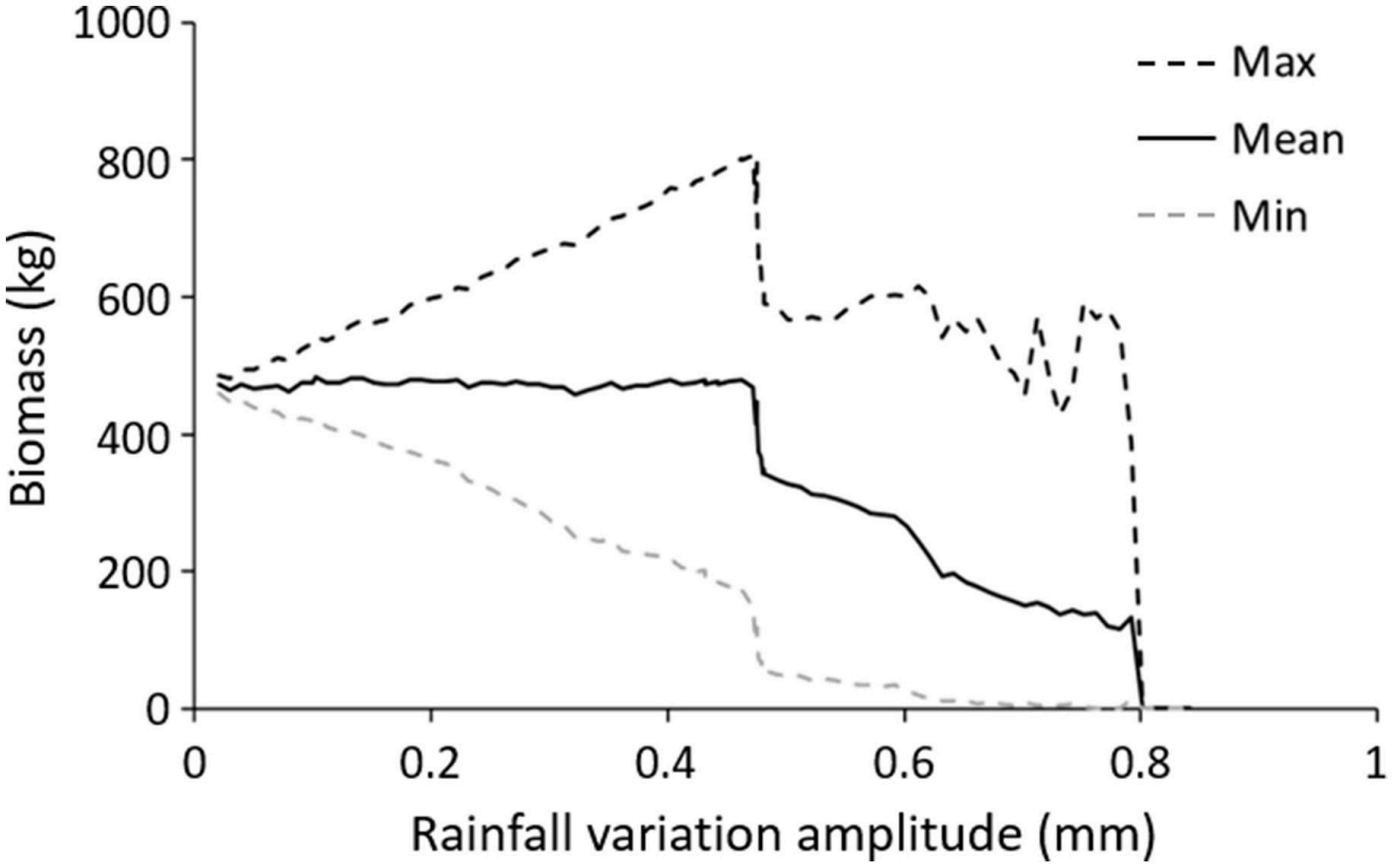
**Effect of seasonal variations in precipitation**. The mean (continuous line), maximum (black dashed line) and minimum (gray dashed line) value of the total population biomass were computed based on the last 4 years of each 12-year simulation run. The simulations were carried out with changing amplitude of the seasonal rainfall regime (mean rainfall 1.3 mm ± amplitude). At initialization, 250 plants were grouped in 25 patches on a 200 × 200 m space (Default parameter values).

The population reacted differently depending on the magnitude of seasonal changes in precipitations (Figure 6). In consecutive simulations featuring variations up to ±0.45 mm, the mean total biomass of the population (estimated during 4 years after a 8-year warm-up) remained remarkably unchanged, while the amplitude of total biomass oscillations increased with the extent of precipitation variations. This did not prevent the formation of the same spatio-termporally stable labyrinth pattern observed with constant water 1.3 mm precipitation. However, the width occupied by the vegetation bands was comparatively narrower in the dry season and larger in the wet one.

**Figure 7:**
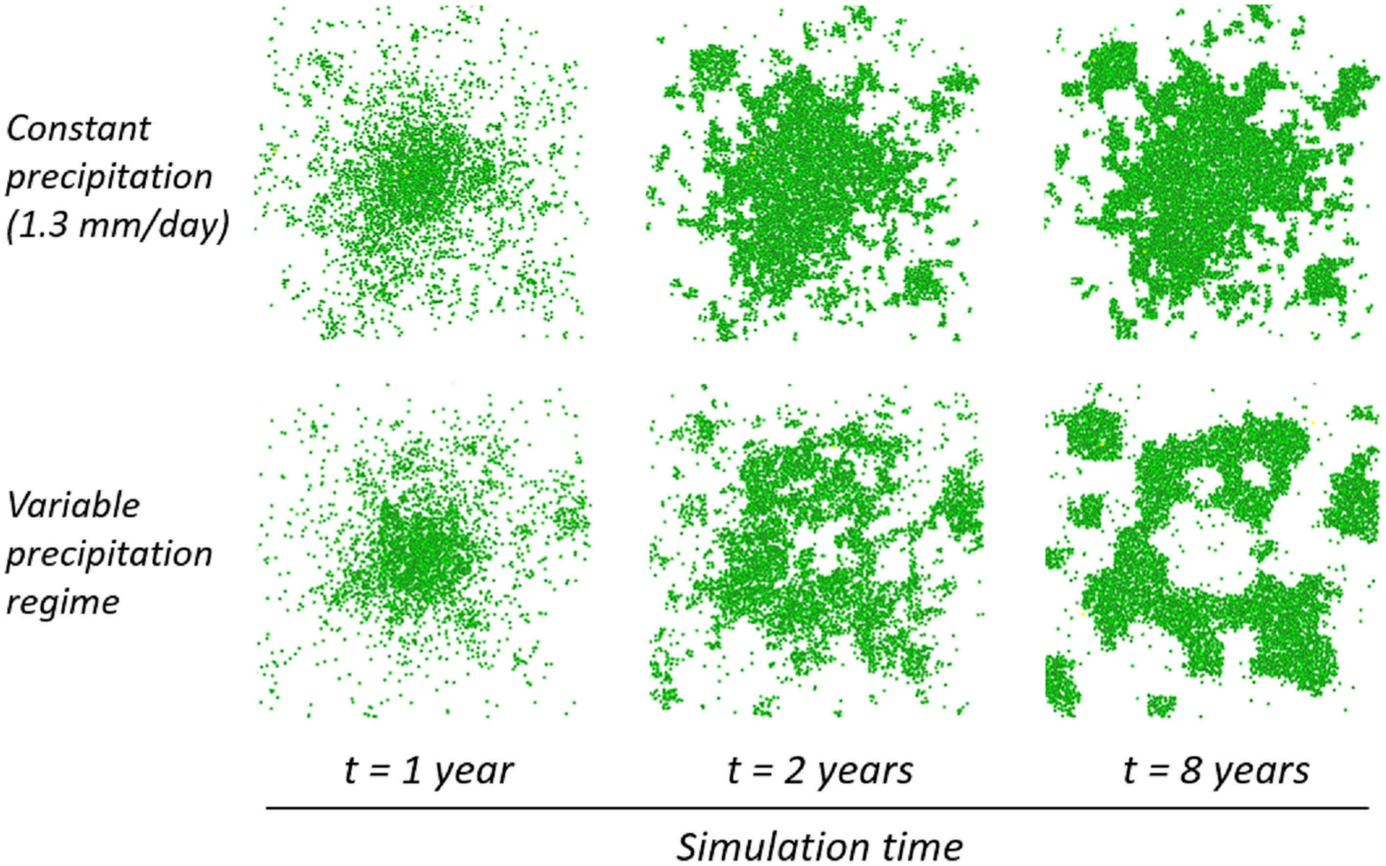
**Spatial dynamics of vegetation under different precipitation regimes**. One patch of 10 plants was simulated on a 50 × 50 m plot. **Top**: formation of an aggregated patch under constant conditions. **Bottom**: emergence of a ring-shaped patch under variable precipitation (sine function varying between 1.0 and 1.6 mm/day with a period of 1 year). (μ = 5 m, Δ*x* = 1 m; other parameters see Table 1).

Under such low seasonal variations (ca. ±0.3 mm) and only under advantageous stochasticity favoring the development of a roughly circular shape, patch borders, while fluctuating with seasons, would keep extending. After 2 years, a clear growing ring pattern would then appear (Figure 7). Following the establishment of the ring, bands would start growing from the latter, which would gradually expand into a helix-like pattern (see Video 2). Understanding the triggers of this phenomenon, which would require further analysis, was beyond the scope of this paper. We conjecture here that this could perhaps be linked with increasing water stress in the center of the patch. This process was observed to follow the installation of a soil water gradient inward the patch (see Datasheet S1.5), and seemed to require that the patch size be large enough comparatively to the surface water diffusion constant. Based on the limited number of runs that were dedicated to the study of this pattern, the ring formation process seemed to be enhanced by a low *D*_*O*_∕*D*_*W*_ ratio (e.g., it was starker and faster to appear with a ratio of 5/0.01 than 10/0.01 for instance). While none of the runs that we performed with constant rainfall gave rise to ring formation, a systematic exploration of the whole parameter space would be required to rigorously establish a solid link between rainfall variability and this processs.

Subjected to more severe seasonal cycles (between ±0.45 and ±0.8 mm), mean total biomass was negatively affected by the intensity of precipitation variations (*y* = –801083x + 730639 *R*^2^ = 0.96). In simulations performed within this range, the population underwent different dynamics starting with an episode of chaotic demographic behavior in which intense growth periods preceded near-extinction declines in a series of periodic oscillations of unpredictable amplitudes. Stable oscillations eventually could occur only after ca. 3000–4000 days. At this point, total biomass and plant numbers would oscillate in a regular fashion, with a low and high season reflecting the seasonal changes in water availability. A 2-year limit cycle then emerged in which the population's spatial distribution followed an alternating usage of the space. On even years, one contiguous half of the plot would be occupied, whereas on odd years, the other would be (see Video 3 of this phenomenon).

Overall, due to the soil's buffering capability, the total biomass cycles were lagging shortly behind the rainfall changes as in Experiment 2A. Seasonal variations in precipitation clearly influenced total plant biomass as well as the shape and amplitude of the peaks (Datasheet S1.6, bottom). Interestingly, it should be noted that initial condition in plant distribution, which affected the shape of patterns under constant rainfall (Figure 2), seemed to have no effect under the cyclic rainfalls featured in Experiment 2B (Datasheet S1.7).

## Discussion

### Ecological results

#### Patterns and individual-scale processes

Simulations were capable of reproducing many patterns visible in nature (Figure 2). They also made it possible to deduce some characteristics of plant populations subject to self-organization and spatial patterning. Plant attributes, which could be studied with this hybrid approach, proved important drivers of patterning. For instance, low seed dispersal distance favored the appearance of clear patterns, whereas high reproductive age aggregated plants in patches (Figure 4).

Spot patterns were known from previous studies to exhibit decreasing biomass from the center of patches (Rietkerk et al., 2002). This was confirmed by the present study, in which plant age also proved to follow the same behavior during the colonization phase (Figure 2B). However, the model also showed that, contrary to biomass density, mean plant biomass increased with distance from the patch center, with the highest biomasses encountered on patch periphery. In bi-dimensional space, biomass hotspots were also visible within the patch, which strongly diverges from the uniform biomass gradient reported with diffusive models (Rietkerk et al., 2002). These actually represent colonization stepstones that provide interesting information about the substructure of vegetation spatial patterns and the dynamics of their formation.

#### Importance of climate variability

The organization in patterns enabled plants to survive in very dry areas, which would be fatal to randomly distributed populations. This spatial organization also seemed to act as a shielding mechanism against disturbances in precipitation, which are common in arid regions. A higher resistance to climate shifts was demonstrated in self-organized populations (see Section Precipitation Shift). In such cases, vegetation suffered a demographic drop and a change of spatial pattern as already hypothesized in a previous study (Rietkerk et al., 2004, see Table 1 esp.). As long as this switch could occur in the short period before total extinction of the vegetation, the population would survive. Interestingly, the initial distribution of plants proved critical in its ability to respond to environmental changes. More generally, as described hereafter, the new precipitation regime imposed new requirements in terms of patch size and population structure. Therefore, all factors influencing patch size, and thereby its age and biomass structure, influenced the population's capacity to react and adapt.

New insights could be gained on the internal structure at the plant level of populations facing water stress. Populations suffering from low rainfall exhibited a high number of seedlings. Surprisingly though, mean individual biomass was also significantly higher than in a populations subject to a more humid environment (Figure 3). This could be explained by the fact that under high water stress there are fewer individuals resulting in a lower plant density. The individuals that survive are thus bigger on average since they are less subject to inter-individual competition (see Datasheet S1.3). Besides, brutal droughts affected most strongly certain categories of plants (i.e., mid-aged plants and plants with medium biomass survived best; Figure 5A). Spatially, isolated seedlings and, under low surface water flow especially, plants in small patches or surrounded by a large number were prone to elimination. This suggests that a minimal patch size was necessary to survive the shift, yet overly large patches would see their central plants most vulnerable. Under seasonal variations and favorable stochastic conditions, the same mechanism proved to lead to the formation of fairy rings (or fairy circles, depending on hydrological parameters) (Figure 7). This recalls results by Sheffer et al. (2011) with a model of aggregated vegetation based on a system of non-linear partial integro-differential equations, and provides an alternative explanation for this spatial pattern, which has also been conjectured to stem from destabilization of a vegetation spot by outward clonal expansion (Sheffer et al., 2007), lateral root-augmentation feedback (Getzin et al., 2015), top-down control by the physical landscape template (Sheffer et al., 2013), and recently, plant-soil negative feedback (Bonanomi et al., 2005; Cartenì et al., 2012; Mazzoleni et al., 2015). Noteworthy is the progression of the biomass front after the appearance of the ring pattern, which has not been the focus of much attention. Instead of simply growing while maintaining shape, or breaking into waves (Cartenì et al., 2012; Marasco et al., 2014), we obtained here a non-isotropic growth forming empty branches leading ultimately to an unusual helix shape (Video 2).

#### Common points and divergences with diffusive models

There were interrogations on whether anterior models able to reproduce vegetation patterns were dependent on their representation of biological dispersal as simplistic physical diffusion (e.g., Okubo, 1980; Rietkerk et al., 2002). The theory by which vegetation patterns could be explained by competition for soil water relied on these models, and it was unclear whether it would resist a more realistic implementation of plant dispersal. The hybrid model confirmed that this behavior is not an artifact of simplified biomass diffusion. The patterns output by diffusive models were on the whole relatively close to the hybrid model output. Consequently, it strengthens the idea that the interplay between differential water availability and plant turnover may represent a sensible cause for patterns reported in the field.

The design of the model allowed for the reproduction of patterns observed in nature while waving some assumptions about the system inherent to previous models. For plant diffusion, space was not considered to have reflecting boundary conditions as in Rietkerk et al. (2002). Seeds dispersing outside of the maps were simply lost, generating an outflow from the system, which is normally not foreseen in mean field models (Kéfi et al., 2007). Besides, the dispersal kernel here was isotropic (sc. it spread seed in all directions equally). All neighbor parcels within a given distance could then be colonized, and the biomass flow was not confined and proportional to biomass deprivation in neighboring cells. This confirms that the shapes of the patterns are not dependent, at least for biomass diffusion, on Euclidean neighborhood nor on directed and targeted colonization of empty soil, which is considered in all the models following Rietkerk et al. (2002). Moreover, no direct interaction happened between plants, as well as no other indirect interaction than competition for water. Especially, unlike in Manor and Shnerb (2008), no density-dependent mortality was introduced in the model. Although the former is an ecologically valid assumption (Harms et al., 2000), considering it in a model that intends to investigate the cause for pattern formation tends to cast doubt on which negative feedback—competition for water or density-dependent mortality—is the source of this phenomenon in the simulations. For the sake of realism and performance, we included a biomass density threshold *K*_*a*_, yet it did not limit total biomass but seedling establishment and was never necessary to reproduce the results presented in this study. Simulations presented in Figure 4 show that longer dispersal distances seem to disrupt pattern formation, which is also consistent with the behavior of reaction-diffusion models in which the biomass diffusion coefficient must be significantly smaller than the water diffusion coefficient to allow the emergence of spatial patterns.

Divergences between the hybrid and diffusion model were still noticeable in the simulation results. While the diffusion models seem to reproduce perfectly the final equilibrium state of the vegetation biomass, it remains questionable whether they also render accurately the pattern formation process. One apparent divergence concerns the patterns, which are less smooth and orderly with the hybrid model. For example, in the results reported in Figure 2, it is clear that patterns are more disordered and less regular with the hybrid model. This can be explained by the fact that when plants start dispersing, they group in high-density patches before trying to spread farther. This creates roughly concentric and then directional dispersal fronts, which have to fight against the increasing shortage of water on their edges when growing in surface and penetrating into “isolated” areas. This differs from the case of diffusion, in which the whole processes are smoothed out by the fast and homogeneous balancing performed by the continuous lateral flows of vegetation. However, the general compatibility between this tendency to osmosis among neighboring parcels, even though rudimentary and questionable, and the mechanisms involved in plant dispersal also explains the relative success of the reaction-diffusion approach.

Another important difference between the hybrid and classical reaction-diffusion approach is that the latter does not allow to properly simulate seasonal variability in rainfall. This comes from the fact that biomass “diffusion” is not coupled with growth. For this reason the diffusion process continues even in the absence of available water. This assumption constitutes one of the main limitations of the PDE models since precipitation events in arid and semi-arid environments are far from being evenly distributed throughout the year.

Unlike previous aggregated deterministic models, the individual-scale stochastic hybrid model proved also very sensitive to initial conditions. For instance, when it was run with plants grouped in 1% of the parcels (i.e., “aggregated distribution”), the population had higher chances of surviving shifts in precipitation (cf. Section Precipitation Shift). The shape of the final patterns varied also visibly between the runs starting with the two initial conditions (Figure 2A). Most importantly, plants were less sensitive to climatic shifts under aggregated distribution.

### On the modeling of vegetation

#### Modeling flexibility

The hybrid solution advanced here proved advantageous in several aspects. It enabled the integration of various facets of vegetation patterning after the first studies of Lefever and Lejeune (1997); HilleRisLambers et al. (2001), and then Rietkerk et al. (2002) increased accuracy of the models by considering more realistic ecological and hydrological processes. The same line of reasoning was followed here by improving the representation of vegetation. In this aspect, the hybrid model allowed the integration and study of plants instead of aggregated vegetation. Plant life cycle could be simulated at the same time as plant-soil interactions. Water dynamics inside the soil could be rendered in an intuitive and exhaustive manner to gain understandability and accuracy. Finally, biological dispersal was represented as realistic seed dispersal instead of continuous diffusion. Practically, the representation of the individuality of plants was critical and made possible the collection of data that were obfuscated by previous models. For instance, spatial patch structure and population age/biomass structure could be analyzed (Figures 2B, 3 respectively). These measurements could allow for valuable individual-scale comparison with data collected in the field.

The hybrid solution could reproduce this complex system made of several components using a relatively simple modular structure with communicating submodels based on an intuitive conceptual framework (Vincenot et al., 2011) taking advantage of the respective capabilities of the SD and IBM paradigms (Figure 1). Plants with their individual characteristics (i.e., algorithmic life cycle based on metabolic processes with relevant state variables such as age or biomass) were naturally integrated inside of IB individuals. Likewise, the hydrology of soil parcels was simulated. SD-IB hybrid modeling made it possible to couple submodels computed in continuous time with other submodels taking decisions in discrete time. This technical capability increased the accuracy of the model by representing processes more naturally. Metabolism and local water dynamics are obviously biological and physical processes that happen continuously. On the other hand, the plant's life cycle and seed dispersal include temporally punctual phenomena and should be modeled in discrete time.

Lastly and perhaps most importantly, the hybrid approach may benefit future works by allowing the fusion of two working hypothesis about the origin of pattern formation as direct inter-individual interactions (Lefever and Lejeune, 1997; D'Odorico et al., 2006) or vegetation-soil interactions (HilleRisLambers et al., 2001; Rietkerk et al., 2002). The present model features both plant individuals and simulates competition for groundwater, making it possible to integrate processes related to both aspects (e.g., seed dispersal and water infiltration respectively). Performing such a task would be challenging with an equation-based approach, due the lack of notion of individual heterogeneity and discrete spatiotemporal interaction. With agent/individual-based modeling, building and analyzing such a model would have been equally hard. While IBM has been extremely helpful in studying plant patterns especially thanks to its ability to represent plant life cycles (e.g., Wiegand et al., 1995; Peters, 2002; Cipriotti et al., 2012), the absence of a way to represent continuous processes (e.g., metabolism, soil hydrology) in an understandable manner and an appropriate computation engine push modelers toward simplifications. Examples of these were given in the introduction about Paruelo et al. (2008) and Lohmann et al. (2012). Cellular automata (CA) approaches, which have been a popular tool to study vegetation spatially (for reviews, see for example Mauchamp et al., 2001; Thiéry et al., 2001), carry the same limitations, and often supplementary ones in their reliance on a fixed discrete grid to represent plants (or aggregated vegetation, in which case individuality is lacking; e.g., the TIGRFLUX model in Thiéry et al., 2001), the static nature of cell interconnections (Hogeweg, 1988), and the synchronicity of cell update (except in asynchronous cellular automata; Fatès, 2013).

The hybrid approach therefore carries the potential for valuable developments in the integration of both ecohydrological dynamics as well as plant-scale mechanisms involved in intra- and interspecific interactions.

#### Model limitations and caveats

The model introduced here allowed for the inclusion of individual plants in simulations. Nonetheless, some limitations remained in the modeling of plant-soil interactions. Plants only draw water on the parcel on which they lie. In high-resolution simulations with small parcels (e.g., 1 × 1 m), it would not be possible to account for root growth on neighboring parcels. It is probably marginal in the case of pattern-forming vegetation like shrubs withal. Future evolutions of the current model could rely on the application of reference case 3b (cf. Section 5.3.2 in Vincenot et al., 2011) for this purpose. Also, while the uptake of water and the increase in biomass could be modeled for each plant, we followed a generic description for these processes, omitting for example root-shoot allocation and other precise biological mechanisms. The same happened for plant dispersal, which was rendered as a single process aggregating seed production, biological dispersal (ballistic, by animals, or other vectors), seed development, seedling viability. All these aspects are not present in the models described so far, but could be well integrated in the frame of the SD-IB hybrid approach.

The added flexibility provided by hybrid modeling comes with a need for greater caution during the design and implementation phases. The description of hybrid models in general is non-trivial because they couple contrasted paradigms. The ODD protocol (Grimm et al., 2010) offers a descriptive framework for agent/individual-based models, which can be easily adapted to hybrid models as done in the present paper with the addition of information on the modeling paradigms used (e.g., time clock type), the implementation platforms for the various submodels, and the mechanisms used to link them (cf. Section Practical Implementation and Fusion of the SD and IB Model Components). Points of linkage between submodels (what we called “hooks” here) should especially be correctly mentioned and discussed. Still, unspoken implementation details may affect model coupling, and we therefore recommend for model descriptions used during the design phase to be very exhaustive and leave as little room as possible for implementation freedom. In most cases, implementation of hybrid models is not a user-friendly process because it consists in the interfacing between existing dissimilar systems. A good perception of program flow and possible coding traps (e.g., race conditions, numerical approximations, defect criticality) is required and this task should therefore better be trusted to seasoned programmers. Modelers should also be aware that different modeling paradigms may come with different representations of time and space. Differences in the former that are not properly handled can generate timing or synchronization issues, numerical inaccuracies, lags and hysteresis, and pathological oscillations. Numerical errors can also come along with divergent spatial resolutions that are not accounted for by design. An interesting example could take place in the present model, in which the soil layer is discretized in a grid of parcels. If the resolution were extremely fine-grained, a single seedling, which is here a discrete point in space, would translate into an exaggeratedly high biomass density on the parcel that it occupies, thereby impacting negatively its own growth through the water uptake process. This would indeed result in an underestimation of the plant's growth rate due to unrealistic water stress.

A simplification potentially detrimental to all individual-based plant models in general as regards seed dispersal was discovered while working with this model. For performance reasons, it may seem appropriate to preemptively not establish a seedling in a parcel whose conditions (here, water availability) would not support its growth. This approach was chosen by Paruelo et al. (2008) among others. We tested the effect of this shortcut by calculating the expected growth rate of the seedling at the time of dispersal and implanting it only if this rate was positive. This approach indeed significantly sped up the simulations by limiting the high number of doomed seedlings that need to be instantiated and thereby require computational resources in vain. However, it also affected to some extent pattern formation (see Datasheet S1.8). This can be explained simply by the fact that, while the estimation of the growth rate at time t may indicate that the seedling won't be able to grow, it ignores future conditions at time t+1 onwards, which can be changed by abiotic (e.g., water diffusion) or biotic (e.g., concurrent establishment of other plants favoring higher water infiltration) factors. Therefore, we recommend caution when choosing whether and with which threshold to implement preemptive seedling removal.

### Implications for future research

The results obtained second the hypothesis that plant-soil dynamics and differential water availability in the soil can be the cause of vegetation patterns. Favoring explicit plant dispersal over a physical biomass diffusion process did not engender significantly discrepant observations (e.g., absence or temporal instability of patterns within the same parameter space, radically different biomass density distribution within patches) that could have cast a doubt on this theory. Still, some divergences could be noticed in the way plant formation took place. The stochastic nature of plant growth and dispersal generated more disordered patterns than what was previously observed with diffusive models. The colonization process also was different. This suggests that interesting behaviors may surface in follow-up studies. Seed dispersal for instance has already proven to be critical to explain peculiar vegetation dynamics in field experiments (Aguiar and Sala, 1997). The hybrid model presented here could be useful in supporting future virtual investigations on the effect of this process (e.g., impact of dispersal kernel shape). Additionally, this model made the collection of data about individual plants achievable. This is a definitive asset, which can certainly give rise to new types of experiments to relate simulations to field data at the individual-scale, and permit ultimately to further substantiate, fine-tune, or on the contrary perhaps falsify the working theory of Rietkerk et al. (2002). Other concurrent hypotheses explaining the formation of vegetation patterns (e.g., particular rainfall regime; Ursino and Contarini, 2006) could also be tested as well, since deterministic or stochastic process related to plant or water dynamics—including direct competition—can be implemented inside of a hybrid model.

As pointed out by Xu et al. (2013), models currently have a “limited ability to predict vegetation dynamics under water stress.” This was rigorously demonstrated through a comparison between model output and empirical data (Powell et al., 2013). Xu et al. (2013) pushed the analysis further and stressed the modeling limitations that would need to be addressed to improve models. The four key components that they highlighted related to plant biophysics, namely the effects of drought on photosynthesis, on plant functionality, on respiration, and on the timing and extent of plant death/regrowth. These aspects, especially the latter one, act at a fine scale and their accurate modeling would therefore benefit from an individual-based hybrid approach capable of integrating plant biophysics with spatial diffusion of water and nutrients. Moreover, the usefulness of having mechanistic processes integrated into physiology models has already been demonstrated (McDowell et al., 2013).

For the sake of illustrating the use of SD-IB hybrid modeling, some assumptions and best guesses were made in this study especially about biology of the plant taxa present at the different locations in which pattern formation occurs. Collecting more precise information on the vegetation type subject to this phenomenon (as done in Barbier et al., 2008) and validating model results against field data (as done in Powell et al., 2013), would allow producing specific simulations for localized cases. This could lead to interesting comparative studies, which could assess through the model potential differences between each case and hopefully thereby contribute to the validation of a governing theory.

## Author contributions

All authors listed, have made substantial, direct and intellectual contribution to the work, and approved it for publication.

## Notes

For demonstration purpose, a simplified version of the model presented in this paper has been released online at the following address: http://christian.vincenot.biz/models.html.

### Conflict of interest statement

The authors declare that the research was conducted in the absence of any commercial or financial relationships that could be construed as a potential conflict of interest.

## Supplementary material

Datasheet S1 **Supplementary data (Figures 1–8)**.

Video 1 **Simulation showing the formation of a labyrinth pattern**. Plants were initially scattered randomly in space, and precipitation remained constant at 1.3 mm. Water density in the soil is visible in the background through a color gradient (black to white for increasing values).

Video 2 **Simulation showing the formation of a vegetation ring, ultimately resulting in an expansion into elongated empty branches forming a star/helix pattern**. Plants were initially grouped in a single patch, and precipitation varied seasonally (See Section Seasonal Variations for details).

Video 3 **Simulation showing the limit cycle reached by vegetation when facing high seasonal variations in precipitation (between 0.5 and 2.1 mm)**.
