# Supplementary material for: Spatial Self-Organization of Vegetation Subject to Climatic Stress—Insights from a System Dynamics—Individual-Based Hybrid Model
Source: Front Plant Sci. 2016 May 24;7:636. doi: 10.3389/fpls.2016.00636 (PMC4877523; doi:10.3389/fpls.2016.00636)
Supplement: Datasheet S1 — Supplementary data (Figures 1–8). [file DataSheet1.PDF]

Supplementary Material S1

Spatial Self-Organization of Vegetation Subject to Climatic Stress – Insights from a System Dynamics - Individual-based Hybrid Model

Christian Ernest VINCENOT[1]*, Fabrizio CARTENI[2], Stefano MAZZOLENI[2], Max RIETKERK[3], Francesco GIANNINO[2]

*** Correspondence:** Christian Vincenot, Christian@Vincenot.biz

1 Advantage of the System Dynamics representation

System Dynamics (SD) makes use of the so-called stock and flow representation. This provides an intuitive way to quickly visualize the continuous relationships between processes and build causal loop diagrams to identify the feedback loops driving the system. The dynamics of the system at runtime is also intuitive as visible in the figure below. For instance, it is conspicuous here from the red-colored flow that water is globally diffusing out of the soil parcel considered (i.e. diffusionSurfaceWater < 0).

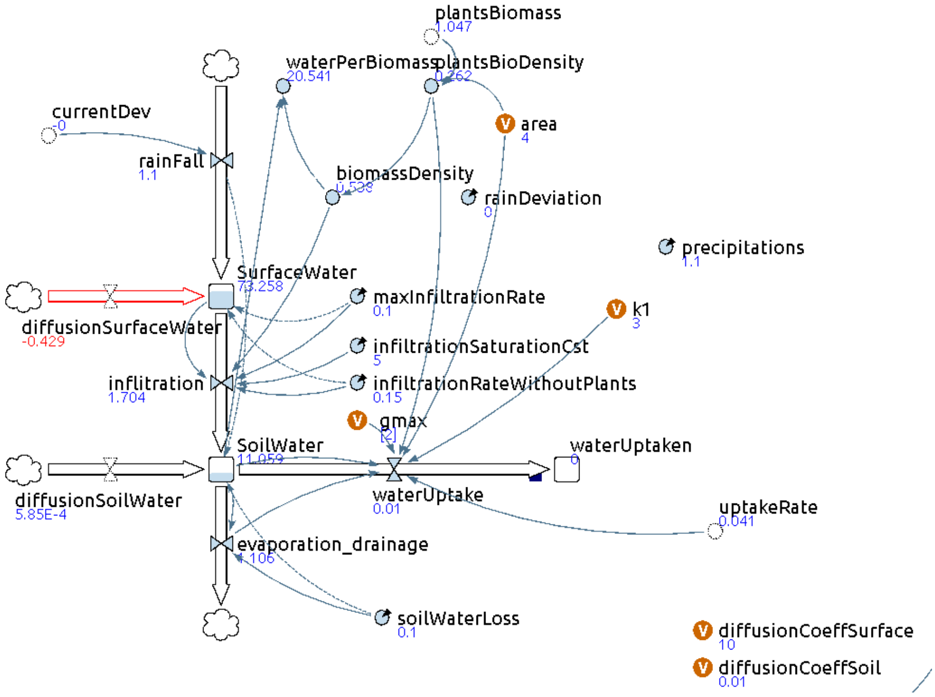

Supplementary Figure 1. System Dynamics (SD) visual display of hydrological processes taking place in one soil parcel.

2 Effect of initial conditions (IC) in plant spatial distribution on pattern formation

The less plants are grouped at initialization, the thinner the labyrinth bands and the corridors.

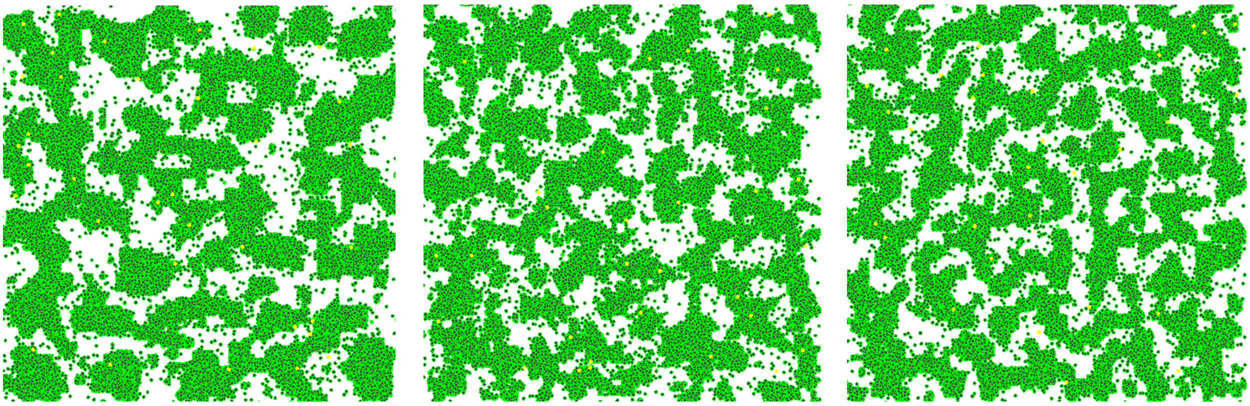

Supplementary Figure 2. Initial conditions (IC) in plant spatial distribution were varied from grouping plants in 100 patches (left), 400 patches (middle), or randomly spreading them on the plot. The simulation was run over 30 years to comfort the results. (Default parameters used. Please refer to Table 1 in the manuscript.)

3 Seed dispersal and average individual biomass

The increase in mean individual biomass with decreasing precipitation could be explained as follows. When precipitation is increased, more water becomes available and supports a larger biomass. This was verified in the model. This biomass can be decoupled in more plants or a higher mean individual biomass or both. Here, we actually observed the former, but not the latter. On the opposite, individual biomass decreases. This comes from the fact that seed production is proportional to biomass in this model. Consequently, an increase in total biomass automatically triggers an increase in number of seeds and a higher competition for suitable space (i.e. soil parcels with enough water). Therefore, plant density increases while mean plant biomass decreases. We performed a small experiment to show how seed productivity impacts population structure. A plant population with same parameters as in Figure 3 was initialized and run 4000 days with seedling production rate 0.001. The latter was then halved and the model run for 2000 days. Finally, it was reduced to 0.0001 for 2000 days. The run showed that, without any change in precipitation, these shifts triggered small changes in patch size, and most importantly an increase in mean plant biomass (from 30.4, to 36.9, and finally 61.5 g) and in the position of the 2nd biomass peak. This seems to support the hypothesis that, under increasing precipitation, the inscrease in number of seeds—coupled with the higher dispersal within patches coming with the increase in area of the latter—is responsible for the decreased individual biomass.

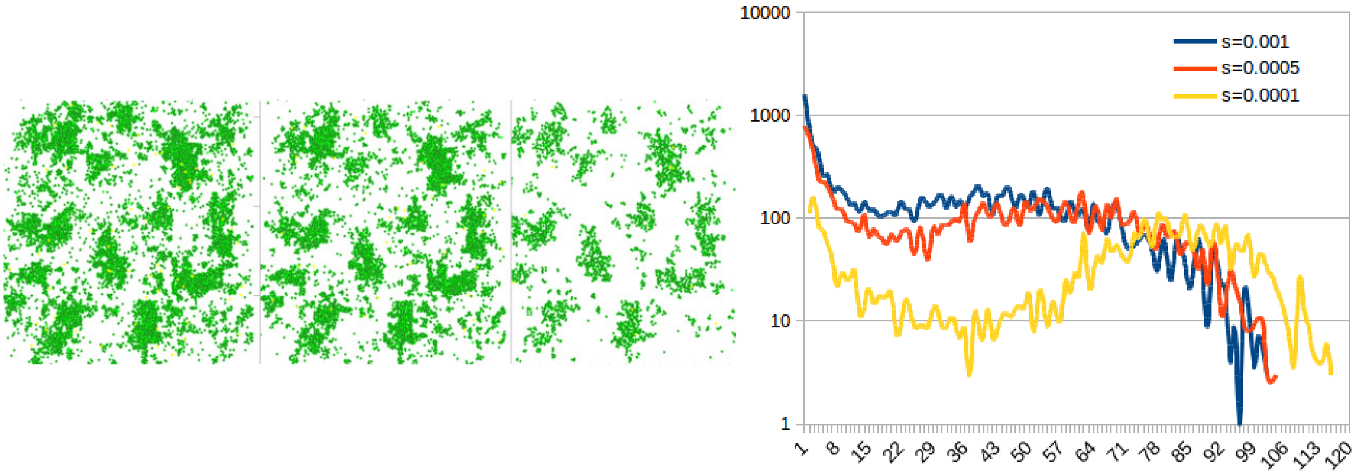

Supplementary Figure 3. Changes in spatial plant cover (left) and individual plant biomass distribution (right) of a population whose plants have consecutively a seed production rate of 0.001, 0.0005, and 0.0001. Shifts were operated after 4000 days and 6000 days.

4 Influence of reproductive age on vegetation patterns

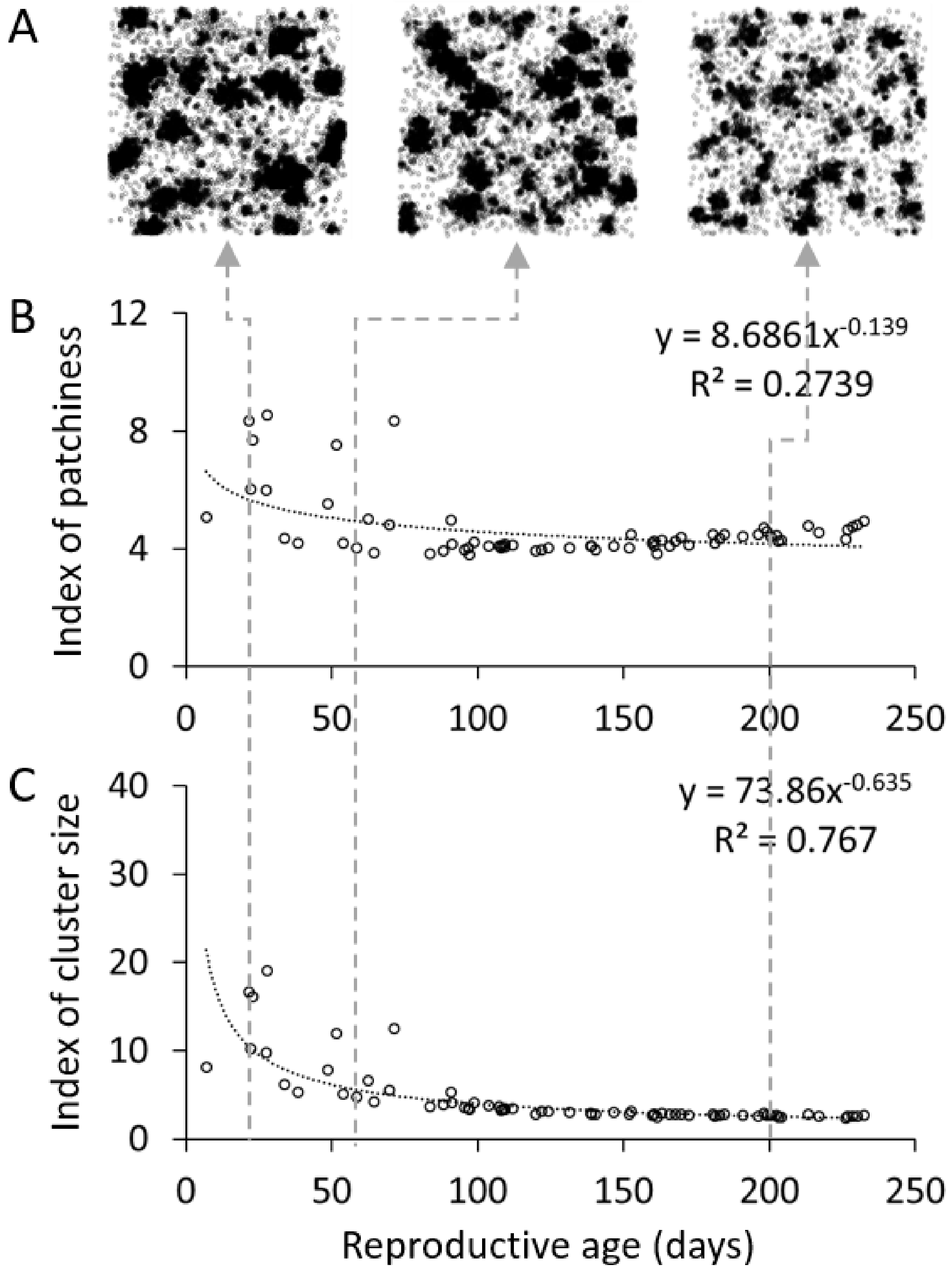

Supplementary Figure 4. The top panel (A) illustrates typical spatial distributions at the end of simulations with 1, 20, and 80 m mean dispersal distance (from left to right). Each plot represents the effect of dispersal distance on (B) the index of patchiness and (C) the index of cluster size. At initialization, 250 plants were grouped in 25 patches on a 200x200 m plot and the experiment was run over 5 years**.** (Default parameter values.)

5 Mechanism of ring formation

The formation of ring patterns could be observed with this model under a regimen of cyclic annual variations in rainfall. The emergence of an area of bare soil in the center of the patch followed the expansion of a soil water density gradient across the patch as visible in the figure below.

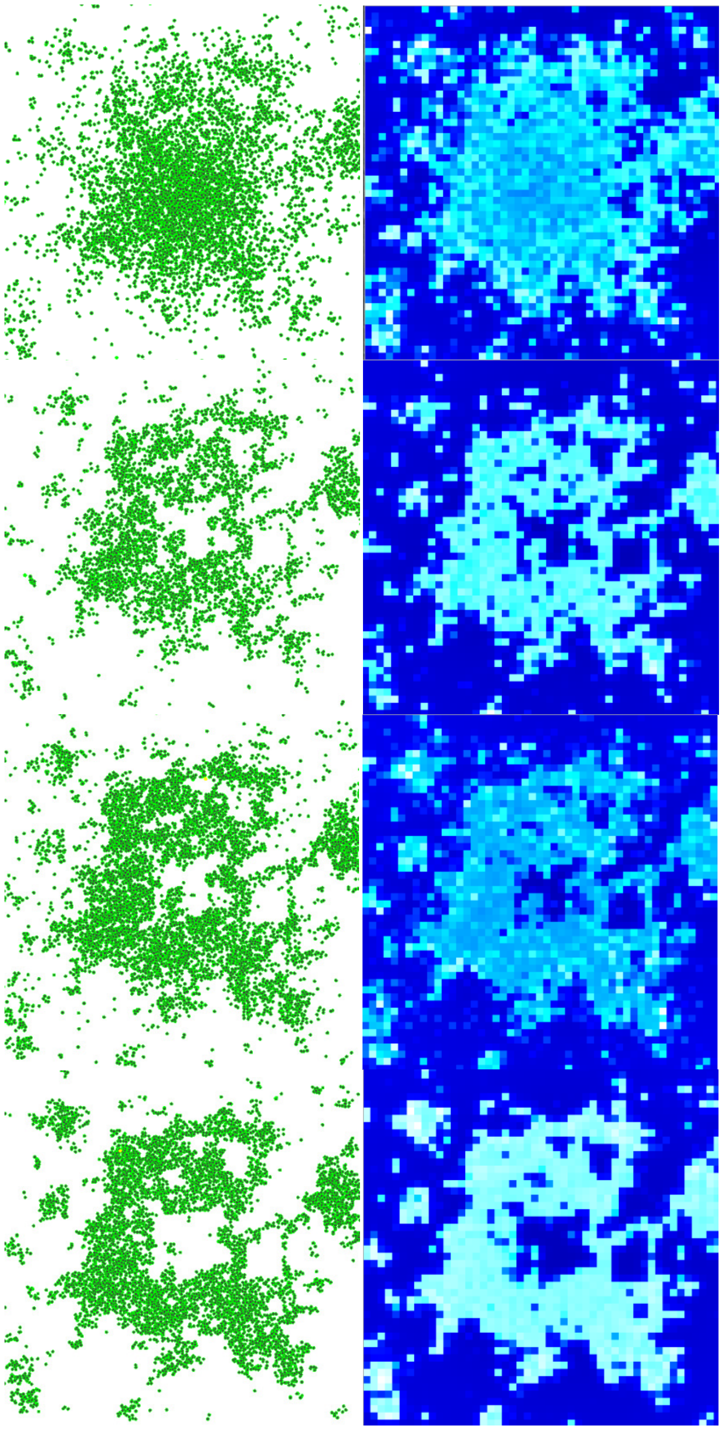

Supplementary Figure 5. Ring formation process with the evolution of plant cover (left) and soil water density (right) after 450 days, 600 days, 750, and 900 days (top to bottom).

6 Effect of seasonal variations in precipitation

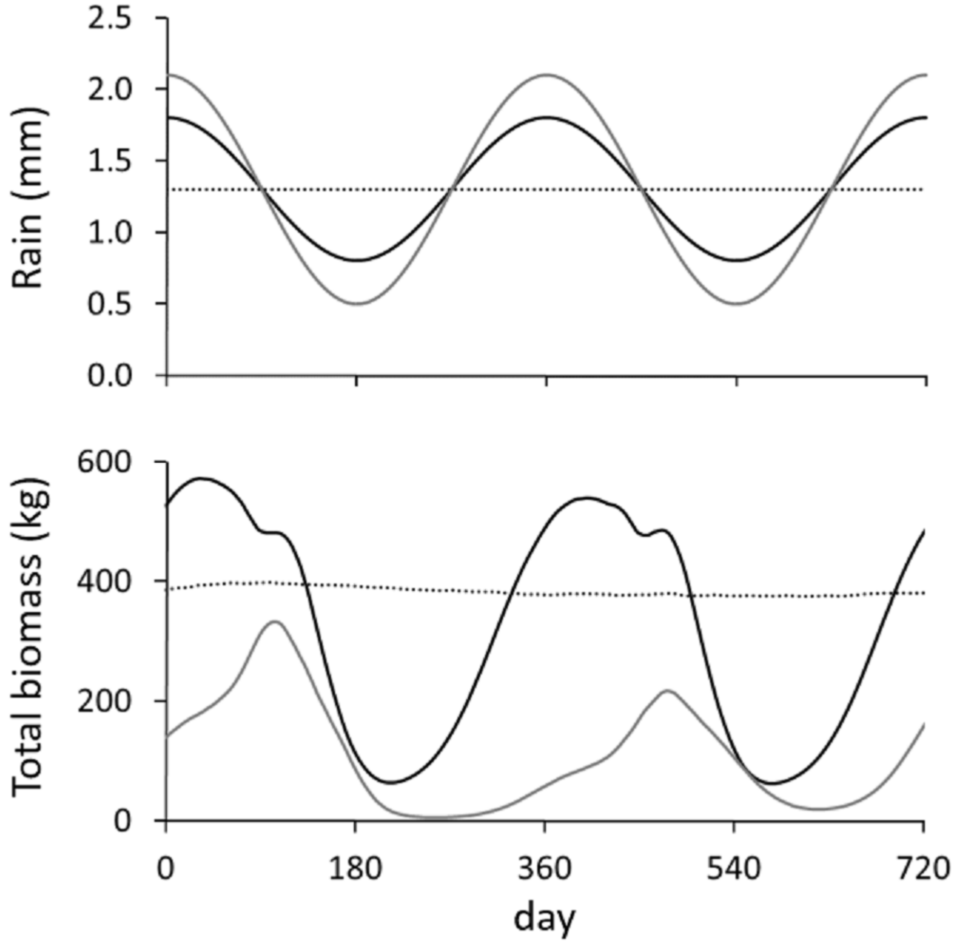

Supplementary Figure 6. At initialization, 250 plants were grouped in 25 patches on a 200x200 m space and the simulation was run with default parameter values over 10 years. The top plot shows the precipitation regimes. The bottom plot shows the daily total biomass associated with the three simulated regimes (each biomass curve style corresponds to the associated precipitation regime). After a period of warm-up leading to stabilization (i.e. 1 year of regular growth for ± 0.5 mm, and up to 5 years of chaotic oscillations for ± 0.8 mm), a limit cycle was reached in both cases. The stronger the variations, the lower the average total biomass. See also Video 3 included as supplementary material.

7 Effect of initial conditions (IC) under seasonal rainfall variations

When cyclic variations in rainfall were simulated, initial conditions (i.e. plants grouped in 25 patches or randomly spread on the map) had no visible effect on final patterns with the parameter set used here (see below). Moreover, the maximum and minimum total biomass reached during oscillations was also unaffected by the IC chosen (i.e. ~70'000 g and ~550'000 g resp. under 1.3 ± 0.5 mm rainfall). The same observation was made as regards the number of plants (i.e. 19'000 and 50'500 individuals resp.).

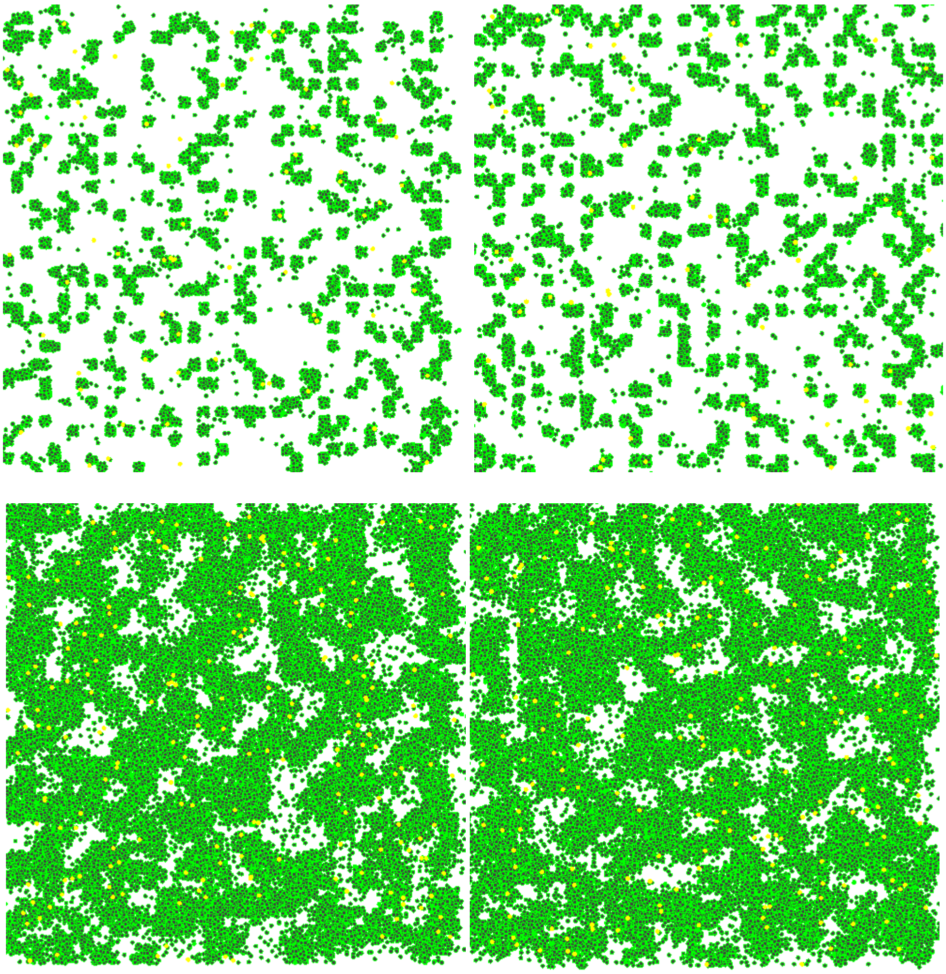

Supplementary Figure 7. Patterns observed in Experiment 2B under medium seasonal variations in rainfall (1.3 ± 0.5 mm) at low (top) and high (bottom) precipitation with random (left) or aggregated IC in plant spatial distribution.

8 Effect of preemptive seed removal

The effect of implementing a seed removal strategy (i.e. not implanting seeds when water condition seem inappropriate for plant growth) to increase performance proved to affect the shape of patterns produced. Below, we show an example of the approximation created on a 200x200m plot. Default parameters were used, except mean dispersal distance, which was increased to 20 m, and seed production rate, which was set to $4x10^{-4}$ to exacerbate the effect.

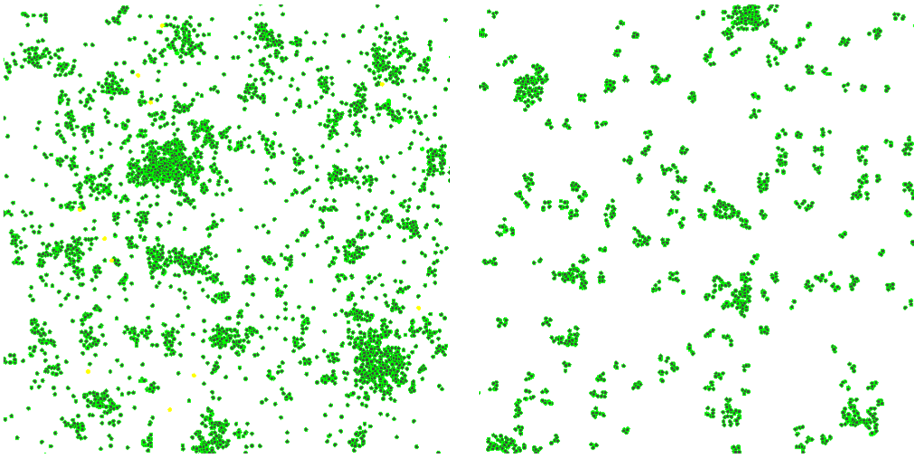

Supplementary Figure 8. The effect of preemptive seed removal on pattern formation. The screenshots presented here are the result of the growth of 2 patches of 25 individuals after 3 years with (left) and without (right) preemptive seed removal.
